# Cross-protection induced by highly conserved human B, CD4^+^, and CD8^+^ T-cell epitopes-based vaccine against severe infection, disease, and death caused by multiple SARS-CoV-2 variants of concern

**DOI:** 10.3389/fimmu.2024.1328905

**Published:** 2024-01-22

**Authors:** Swayam Prakash, Nisha R. Dhanushkodi, Latifa Zayou, Izabela Coimbra Ibraim, Afshana Quadiri, Pierre Gregoire Coulon, Delia F. Tifrea, Berfin Suzer, Amin Mohammed Shaik, Amruth Chilukuri, Robert A. Edwards, Mahmoud Singer, Hawa Vahed, Anthony B. Nesburn, Baruch D. Kuppermann, Jeffrey B. Ulmer, Daniel Gil, Trevor M. Jones, Lbachir BenMohamed

**Affiliations:** ^1^ Laboratory of Cellular and Molecular Immunology, Gavin Herbert Eye Institute, University of California Irvine, School of Medicine, Irvine, CA, United States; ^2^ High Containment Facility, University of California Irvine, School of Medicine, Irvine, CA, United States; ^3^ Department of Pathology and Laboratory Medicine, School of Medicine, the University of California Irvine, Irvine, CA, United States; ^4^ Department of Vaccines and Immunotherapies, TechImmune, LLC, University Lab Partners, Irvine, CA, United States; ^5^ Division of Infectious Diseases and Hospitalist Program, Department of Medicine, School of Medicine, the University of California Irvine, Irvine, CA, United States; ^6^ Institute for Immunology; University of California Irvine, School of Medicine, Irvine, CA, United States

**Keywords:** SARS-CoV-2, SL-CoVs, COVID-19, vaccine, epitopes, antibodies, T cells, immunity

## Abstract

**Background:**

The coronavirus disease 2019 (COVID-19) pandemic has created one of the largest global health crises in almost a century. Although the current rate of Severe acute respiratory syndrome coronavirus 2 (SARS-CoV-2) infections has decreased significantly, the long-term outlook of COVID-19 remains a serious cause of morbidity and mortality worldwide, with the mortality rate still substantially surpassing even that recorded for influenza viruses. The continued emergence of SARS-CoV-2 variants of concern (VOCs), including multiple heavily mutated Omicron sub-variants, has prolonged the COVID-19 pandemic and underscores the urgent need for a next-generation vaccine that will protect from multiple SARS-CoV-2 VOCs.

**Methods:**

We designed a multi-epitope-based coronavirus vaccine that incorporated B, CD4^+^, and CD8^+^ T- cell epitopes conserved among all known SARS-CoV-2 VOCs and selectively recognized by CD8^+^ and CD4^+^ T-cells from asymptomatic COVID-19 patients irrespective of VOC infection. The safety, immunogenicity, and cross-protective immunity of this pan-variant SARS-CoV-2 vaccine were studied against six VOCs using an innovative triple transgenic h-ACE-2-HLA-A2/DR mouse model.

**Results:**

The pan-variant SARS-CoV-2 vaccine (i) is safe , (ii) induces high frequencies of lung-resident functional CD8^+^ and CD4^+^ T_EM_ and T_RM_ cells , and (iii) provides robust protection against morbidity and virus replication. COVID-19-related lung pathology and death were caused by six SARS-CoV-2 VOCs: Alpha (B.1.1.7), Beta (B.1.351), Gamma or P1 (B.1.1.28.1), Delta (lineage B.1.617.2), and Omicron (B.1.1.529).

**Conclusion:**

A multi-epitope pan-variant SARS-CoV-2 vaccine bearing conserved human B- and T- cell epitopes from structural and non-structural SARS-CoV-2 antigens induced cross-protective immunity that facilitated virus clearance, and reduced morbidity, COVID-19-related lung pathology, and death caused by multiple SARS-CoV-2 VOCs.

## Introduction

While the Wuhan Hu1 variant of SARS-CoV-2 is the ancestral reference virus, Alpha (lineage B.1.1.7), Beta (lineage B.1.351), Gamma (lineage B.1.1.28), and Delta (lineage B.1.617.2) variants of concern (VOCs) subsequently emerged in the United Kingdom, South Africa, Brazil, and India, respectively, between 2020 and 2022 (1). The most recent SARS CoV-2 variants, including multiple heavily mutated Omicron (B.1.1.529) sub-variants, have prolonged the COVID-19 pandemic (2–6). These new variants emerged beginning December 2020 at a much higher rate, with the accumulation of two mutations per month, and exerting strong selective pressure on the immunologically important SARS-CoV-2 genes (7). The Alpha, Beta, Gamma, Delta, and Omicron variants are defined as VOCs based on their high transmissibility associated with increased hospitalizations and deaths (8). This is a result of reduced neutralization by antibodies generated by previous variants and/or by the first-generation COVID-19 vaccines, together with failures of treatments and diagnostics (9, 10). Dr. Peter Marks, Director/CBER (Center for Biologics Evaluation and Research) for the FDA recently outlined the need for a superior next-generation vaccine that will protect from multiple SARS-CoV-2 VOCs (11, 12).

Besides SARS CoV-2 variants, two additional coronaviruses from the severe acute respiratory syndrome (SARS)-like betacoronavirus (sarbecovirus) lineage, SARS coronavirus (SARS-CoV-1) and MERS-CoV, have caused epidemics and pandemics in humans over the past 20 years (13). In addition, the discovery of diverse sarbecoviruses in bats together with the frequent “jumping” of these zoonotic viruses from bats to intermediate animal hosts raises the possibility of another COVID-19- like pandemic in the future (14–19). Hence, there is an urgency to develop a pre-emptive universal pan-variant SARS-CoV-2 vaccine to protect against all SARS-CoV-2 variants, SARS-CoV, MERS-CoV, and other zoonotic Sarbecoviruses with the potential to jump from animals into humans.

The SARS-CoV-2 genome comprises 29,903 bp. The Spike protein is a predominant surface antigen of SARS-CoV-2 involved in the docking and penetration of the virus into the target host cells (20–22). As such, the Spike protein is the main target of the first-generation COVID-19 subunit vaccines aiming mainly at inducing neutralizing antibodies (23, 24). Nearly 56% of the 10 billion doses of first-generation COVID-19 vaccines are based on the Spike antigen alone (25), while the remaining 44% were based on whole- virion- inactivated (WVI) vaccines (26, 27). Both the Spike-based COVID-19 sub-unit vaccines and the whole virion-inactivated vaccines were successful (20–22). However, because the Spike protein is the most mutated SARS-CoV-2 antigen, these first-generation vaccines lead to immune evasion by many new variants and sub-variants, such as Omicron XBB.1.5 sub-variant (25, 28, 29). Therefore, the next generation of COVID-19 vaccines should also target other highly conserved structural and non-structural SARS-CoV-2 antigens capable of inducing protection by cross-reactive CD4^+^ and CD8^+^ T cells (30–33).

Previously, we mapped and characterized the antigenicity and immunogenicity of genome-wide B cell, CD4^+^ T cell, and CD8^+^ T- cell epitopes that are highly conserved (33). We hypothesize that multi-epitope vaccine candidates that express these highly conserved, antigenic, and immunogenic B- and T- cell epitopes will provide broader global population coverage against multiple SARS-CoV-2 VOCs. The present study (1) identified seven B- cell epitopes, six CD4^+^ T- cell epitopes, and 16 CD8^+^ T- cell epitopes that are highly conserved within (i) 8.7 million genome sequences of SARS-CoV-2, (ii) all previous and current SARS-CoV-2 variants , (iii) SARS-CoV , (iv) MERS-CoV, (v) common cold coronaviruses (HKU, OC1 ), and (vi) in animal CoV (i.e., bats, civet cats, pangolin, and camels); (2) established that those epitopes were selectively recognized by B cells, CD4^+^ T cells, and CD8^+^ T cells from “naturally protected” asymptomatic COVID-19 patients; and (3) demonstrated that a multi-epitope pan-variant SARS-CoV-2 vaccine that includes the above B cell, CD4^+^ T cell, and CD8^+^ T- cell epitopes generated cross-protection against all the five SARS-CoV-2 VOCs, i.e., SARS-CoV-2 (USA-WA1/2020), Alpha (B.1.1.7), Beta (B.1.351), Gamma (P.1), Delta (B.1.617.2), and Omicron (B.1.1.529) in a novel triple transgenic HLA-A*02:01/HLA-DR hACE-2 mouse model of COVID-19.

## Materials and methods

### Viruses

SARS-CoV-2 viruses specific to six variants, namely, (i) SARS-CoV-2-USA/WA/2020 (Batch Number: G2027B ), (ii) Alpha (B.1.1.7) (isolate England/204820464/2020, Batch Number: C2108K ), (iii) Beta (B.1.351) (isolate South Africa/KRISP-EC-K005321/2020; Batch Number: C2108F), (iv) Gamma (P.1) (isolate hCoV-19/Japan/TY7-503/2021; Batch Number: G2126A), (v) Delta (B.1.617.2) (isolate h-CoV-19/USA/MA29189; Batch number: G87167), and Omicron (BA.1.529) (isolate h-CoV-19/USA/FL17829; Batch number: G76172) were procured from Microbiologics (St. Cloud, MN). The initial batches of viral stocks were propagated to generate high-titer virus stocks. Vero E6 (ATCC-CRL1586 ) cells were used for this purpose using an earlier published protocol (34). Procedures were completed only after appropriate safety training was obtained using an aseptic technique under BSL-3 containment.

### Triple transgenic mice immunization with SARS-CoV-2 conserved peptides and infection

The University of California—Irvine conformed to the Guide for the Care and Use of Laboratory Animals published by the US National Institute of Health (IACUC protocol No. AUP-22-086). Seven- to eight-week-old triple transgenic HLA-A*02:01/HLA-DRB1*01:01-hACE-2 mice (n=60) were included in this experiment. Mice were subcutaneously immunized with a pool of conserved Pan-Coronavirus peptides. The peptide pool administered per mouse comprised 25 μg each of the 9-mer long 16 CD8^+^ T-cell peptides (ORF1ab_2210–2218_, ORF1ab_3013–3021_, ORF1ab_4283–4291_, ORF1ab_6749–6757_, ORF6_3–11_, ORF7b_26–34_, ORF8a_73–81_, ORF10_3–11_, ORF10_5–13_, S_958–966_, S_1000–1008_, S_1220–1228_, E_20–28_, E_26–34_, M_52–60_, and M_89–97_), 15-mer long 6 CD4^+^ T- cell epitopes (ORF1a_1350–1365_, ORF6_12–26_, ORF8b_1–15_, S_1–13_, M_176–190_, and N_388–403_), and 9 B-cell peptides. The pool of peptides was then mixed with 25 μg of CpG and 25 μg of alum to prepare the final composition. Mice were immunized with the peptide pool on Day 0 and Day 14 of the experiment. On Day 28, 14 days following the second immunization, mice were divided into six groups and intranasally infected with 1 × 10^5^ pfu of SARS-CoV-2 (USA-WA1/2020) (n=10), 6 × 10^3^ pfu of SARS-CoV-2-Alpha (B.1.1.7) (n=10), 6 × 10^3^ pfu of SARS-CoV-2-Beta (B.1.351) (n=10), 5 × 10^2^ pfu of SARS-CoV-2-Gamma (P.1) (n=10), 8 × 10^3^ pfu of SARS-CoV-2-Delta (B.1.617.2) (n=10), and 6.9 × 10^4^ pfu of SARS-CoV-2-Omicron (B.1.1.529) (n=10). The viruses were diluted, and each mouse was administered intranasally with 20 μl volume. Mice were monitored daily for weight loss and survival until Day 14 p.i. Throat swabs were collected for viral titration on Days 2, 4, 6, 8, 10, and 14 post-infection. The experiment was repeated twice to overcome data bias.

Before beginning the vaccine experiments, we performed a well-curated LD50 experiment in triple transgenic HLA-A*02:01/HLA-DRB1*01:01-hACE-2 mice, for each of the variants, namely, SARS-CoV-2 WA/USA, Alpha (B.1.1.7), Beta (B.1.351), Gamma (P.1), Delta (B.1.617.2), and Omicron (B.1.1.529) variant at 1 × 10^5^ PFU, 8 × 10^4^ PFU, 1 × 10^4^ PFU, 5 × 10^3^ PFU, and 5 × 10^2^ PFU. Based on the LD50 results obtained, the triple transgenic HLA-A*02:01/HLA-DRB1*01:01-hACE-2 mice were challenged with 1 × 10^5^ pfu for SARS-CoV-2 (USA-WA1/2020), 6 × 10^3^ pfu for SARS-CoV-2-Alpha (B.1.1.7) and SARS-CoV-2-Beta (B.1.351), 5 × 10^2^ pfu for SARS-CoV-2-Gamma (P.1), 8 × 10^3^ pfu for SARS-CoV-2-Delta (B.1.617.2), and 6.9 × 10^4^ pfu for SARS-CoV-2-Omicron (B.1.1.529) variants.

### Human study population cohort and HLA genotyping

In this study, we have included 210 subjects from a pool of over 682 subjects. Written informed consent was obtained from participants before inclusion. The subjects were categorized as mild to severe COVID-19 groups and have undergone treatment at the University of California Irvine Medical Center between July 2020 and July 2022 (Institutional Review Board protocol no. 2020-5779). SARS-CoV-2 positivity was defined by a positive RT-PCR on nasopharyngeal swab samples. All the subjects were genotyped by PCR for class I HLA-A*02:01 and class II HLA-DRB1*01:01 among the 682 patients (and after excluding a few for which the given amount of blood was insufficient —i.e., <6ml), we ended up with 210 that were genotyped for HLA-A*02:01^+^ or/and HLA-DRB1*01:01^+^ (35, 36). Based on the severity of symptoms and ICU admission/intubation status, the subjects were divided into five broad severity categories, namely, Severity 5, patients who died from COVID-19 complications; Severity 4, infected COVID-19 patients with severe disease that were admitted to the intensive care unit (ICU) and required ventilation support; Severity 3, infected COVID-19 patients with severe disease that required enrollment in ICU, but without ventilation support; Severity 2, infected COVID-19 patients with moderate symptoms that involved a regular hospital admission; Severity 1, infected COVID-19 patients with mild symptoms; and Severity 0, infected individuals with no symptoms. Demographically, the 210 patients included were from mixed ethnicities [Hispanic (34%), Hispanic Latino (29%), Asian (19%), Caucasian (14%), Afro-American (3%), and Native Hawaiian and Other Pacific Islander descent (1%)].

### Sequence comparison among variants of SARS-CoV-2 and animal CoV strains

We retrieved nearly 8.5 million human SARS-CoV-2 genome sequences from the GISAID database representing countries from North America, South America, Central America, Europe, Asia, Oceania, Australia, and Africa. All the sequences included in this study were retrieved either from the NCBI GenBank (www.ncbi.nlm.nih.gov/nuccore) or GISAID (www.gisaid.org). Multiple sequence alignment was performed keeping SARS-CoV-2-Wuhan-Hu-1 (MN908947.3) protein sequence as a reference against all the SARS-CoV-2 VOCs, common cold, and animal CoV strains. The sequences were aligned using the high throughput alignment tool DIAMOND (37).This comprised of all the VOCs and VBMs of SARS-CoV-2 (B.1.177, B.1.160, B.1.1.7, B.1.351, P.1, B.1.427/B.1.429, B.1.258, B.1.221, B.1.367, B.1.1.277, B.1.1.302, B.1.525, B.1.526, S:677H.Robin1, S:677P.Pelican, B.1.617.1, B.1.617.2, and B,1,1,529) and common cold SARS-CoV strains (SARS-CoV-2-Wuhan-Hu-1 (MN908947.3), SARS-CoV-Urbani (AY278741.1), HKU1-Genotype B (AY884001), CoV-OC43 (KF923903), CoV-NL63 (NC_005831), CoV-229E (KY983587), and MERS (NC_019843)). In addition, for evaluating the evolutionary relationship among the SARS-CoV-2 variants and common cold CoV strains, we have included whole-genome sequences from the bat (RATG13 (MN996532.2), ZXC21 (MG772934.1), YN01 (EPI_ISL_412976), YN02(EPI_ISL_412977), WIV16 (KT444582.1), WIV1 (KF367457.1), YNLF_31C (KP886808.1), and Rs672 (FJ588686.1)), pangolin (GX-P2V (MT072864.1), GX-P5E (MT040336.1), GX-P5L (MT040335.1), GX-P1E (MT040334.1), GX-P4L (MT040333.1), GX-P3B (MT072865.1), MP789 (MT121216.1), and Guangdong-P2S (EPI_ISL_410544)), camel (KT368891.1, MN514967.1, KF917527.1, and NC_028752.1), and civet (Civet007, A022, and B039)).

### SARS-CoV-2 CD8^+^ and CD4^+^ T-cell epitope prediction

Epitope prediction was performed considering the spike glycoprotein (YP_009724390.1) for the reference SARS-CoV-2 isolate, Omicron BA.2. The reference spike protein sequence was used to screen CD8^+^ T cell and CD4^+^ T- cell epitopes. The tools used for CD8^+^ T- cell-based epitope prediction were SYFPEITHI, MHC-I binding predictions, and Class I Immunogenicity. Of these, the latter two were hosted on the IEDB platform. We used multiple databases and algorithms for the prediction of CD4^+^ T- cell epitopes, namely, SYFPEITHI, MHC-II Binding Predictions, Tepitool, and TEPITOPEpan. For CD8^+^ T cell epitope prediction, we selected the 5 most frequent HLA-A class I alleles (HLA-A*01:01, HLA-A*02:01, HLA-A*03:01, HLA-A*11:01, HLA-A*23:01) with nearly 80% coverage of the world population, regardless of race and ethnicity, using a phenotypic frequency cutoff ≥ 6%. Similarly, for CD4^+^ T- cell epitope prediction, HLA-DRB1*01:01, HLA-DRB1*11:01, HLA-DRB1*15:01, HLA-DRB1*03:01, and HLA-DRB1*04:01 alleles with population coverage of 60% were selected. Subsequently, using NetMHC, we analyzed the SARS-CoV-2 protein sequence against all the MHC-I and MHC-II alleles. Epitopes with 9-mer lengths for MHC-I and 15-mer lengths for MHC-II were predicted. Subsequently, the peptides were analyzed for binding stability to the respective HLA allotype. Our stringent epitope selection criteria were based on picking the top 1% epitopes focused on prediction percentile scores. N and O glycosylation sites were screened using NetNGlyc 1.0 and NetOGlyc 4.0 prediction servers, respectively.

### Population-coverage- based T- cell epitope selection

For a robust epitope screening, we evaluated the conservancy of CD8^+^ T cell, CD4^+^ T cell, and B- cell epitopes within spike glycoprotein of Human-SARS-CoV-2 genome sequences representing North America, South America, Africa, Europe, Asia, and Australia. As of 20 April 2022, the GISAID database extrapolated 8,559,210 human-SARS-CoV-2 genome sequences representing six continents. Population coverage calculation (PPC) was carried out using the Population Coverage software hosted on the IEDB platform. PPC was performed to evaluate the distribution of screened CD8^+^ and CD4^+^ T- cell epitopes in the world population at large in combination with HLA-I (HLA-A*01:01, HLA-A*02:01, HLA-A*03:01, HLA-A*11:01, and HLA-A*23:01) and HLA-II (HLA-DRB1*01:01, HLA-DRB1*11:01, HLA-DRB1*15:01, HLA-DRB1*03:01, and HLA-DRB1*04:01) alleles.

### T- cell epitopes screening, selection, and peptide synthesis

Peptide-epitopes from 12 SARS-CoV-2 proteins, including 9-mer long 16 CD8^+^ T- cell epitopes (ORF1ab_2210–2218_, ORF1ab_3013–3021_, ORF1ab_4283–4291_, ORF1ab_6749–6757_, ORF6_3-11_, ORF7b_26–34_, ORF8a_73–81_, ORF10_3–11_, ORF10_5–13_, S_958–966_, S_1000–1008_, S_1220–1228_, E_20–28_, E_26–34_, M_52–60_, and M_89–97_) and 15-mer long 6 CD4^+^ T- cell epitopes (ORF1a_1350–1365_, ORF6_12–26_, ORF8b_1–15_, S_1–13_, M_176–190_, and N_388–403_) that we formerly identified were selected as described previously (33). The Epitope Conservancy Analysis tool was used to compute the degree of identity of CD8^+^ T- cell and CD4^+^ T- cell epitopes within a given protein sequence of SARS-CoV-2 set at 100% identity level (33). Peptides were synthesized as previously described (21st Century Biochemicals, Inc, Marlborough, MA). The purity of peptides determined by both reversed-phase high-performance liquid chromatography and mass spectroscopy was over 95%. Peptides were first diluted in DMSO and later in PBS (1 mg/mL concentration). The helper T-lymphocyte (HTL) epitopes for the selected SARS-CoV-2 proteins were predicted using the MHC-II epitope prediction tool from the Immune Epitope Database (IEDB, http://tools.iedb.org/mhcii/). Selected epitopes had the lowest percentile rank and IC_50_ values. Additionally, the selected epitopes were checked by the IFN epitope server (http://crdd.osdd.net/raghava/ifnepitope/) for the capability to induce Th1 type immune response accompanied by IFN-ϒ production. Cytotoxic T-lymphocyte (CTL) epitopes for the screened proteins were predicted using the NetCTL1.2 server (http://www.cbs.dtu.dk/services/NetCTL/).

### SARS-CoV-2 B- cell epitope prediction

Linear B- cell epitope predictions were carried out on the spike glycoprotein (S), the primary target of B- cell immune responses for SARS-CoV. We used the BepiPred 2.0 algorithm embedded in the B- cell prediction analysis tool hosted on the IEDB platform. For each protein, the epitope probability score for each amino acid and the probability of exposure was retrieved. Potential B- cell epitopes were predicted using a cutoff of 0.55 (corresponding to a specificity >0.81 and sensitivity <0.3) and considering sequences having more than five amino acid residues. This screening process resulted in eight B-cell peptides. These epitopes represent all the major non-synonymous mutations reported among the SARS-CoV-2 variants. One B-cell epitope (S_439–482_) was observed to possess the maximum number of variant-specific mutations. Structure-based antibody prediction was performed using Discotope 2.0, and a positivity cutoff greater than −2.5 was applied (corresponding to specificity ≥ 0.80 and sensitivity <0.39), using the SARS-CoV-2 spike glycoprotein structure (PDB ID: 6M1D).

### TaqMan quantitative polymerase reaction assay for the screening of SARS-CoV-2 variants in COVID-19 patients

We utilized a laboratory-developed modification of the CDC SARS-CoV-2 RT-PCR assay, which received Emergency Use Authorization by the FDA on April 17th 2020. (https://www.fda.gov/media/137424/download [accessed 24 March 2021]).

### Mutation screening assays

SARS-CoV-2-positive samples were screened by four multiplex RT-PCR assays. Through the qRT-PCR, we screened for 11 variants of SARS-CoV-2 in our patient cohort. The variants that were screened included B.1.1.7 (Alpha), B.1.351 (Beta), P.1 (Gamma), and B.1.427/B.1.429 (Epsilon), B.1.525 (Eta), R.1, P.2 (Zeta), B.1.526 (Iota), B.1.2/501Y or B.1.1.165, B.1.1.529 (BA.1) (Omicron), B.1.1.529 (BA.2) (Omicron), and B.1.617.2 (Delta). The sequences for the detection of Δ69–70 were adapted from a multiplex real-time RT-PCR assay for the detection of SARS-CoV-2 (38). The probe overlaps with the sequences that contain amino acids 69 –70; therefore, a negative result for this assay predicts the presence of deletion S-Δ69–70 in the sample. Using a similar strategy, a primer/probe set that targets the deletion S- Δ242–244 was designed and was run in the same reaction with S-Δ69–70. In addition, three separate assays were designed to detect spike mutations S-501Y, S-484K, and S-452R, and wild-type positions S-501N, S-484E, and S-452L.

Briefy, 5 µl of the total nucleic acid eluate was added to a 20- µl total volume reaction mixture (1× TaqPath 1-Step RT-qPCR Master Mix, CG [Thermo Fisher Scientific, Waltham, MA], with 0.9 mM each primer and 0.2 mM each probe). The RT-PCR was carried out using the ABI StepOnePlus thermocycler (Life Technologies, Grand Island, NY). The S-N501Y, S-E484K, and S-L452R assays were carried out under the following running conditions: 25°C for 2 min, then 50°C for 15 min, followed by 10 min at 95°C and 45 cycles of 95°C for 15 s and 65°C for 1 min. The Δ 69–70/Δ242–244 assays were run under the following conditions: 25°C for 2 min, then 50°C for 15 min, followed by 10 min at 95°C and 45 cycles of 95°C for 15 s and 60°C for 1 min. Samples displaying typical amplification curves above the threshold were considered positive. Samples that yielded a negative result or results in the S-Δ69–70/Δ242–244 assays or were positive for S-501Y P2, S-484K P2, and S-452R P2 were considered screen positive and assigned to a VOC.

### Neutralizing antibody assays for SARS-CoV-2

Serially diluted heat-inactivated plasma (1:3) and 300 pfu of SARS-CoV-2 variants are combined in Dulbecco’s modified Eagle’s medium (DMEM) and incubated at 37°C 5% CO_2_ for 30 min. After neutralization, the antibody–virus inoculum was transferred onto Vero E6 cells (ATCC C1008) and incubated at 34°C 5% CO_2_ for 1 h. The cells were then fixed with 10% neutral buffered formalin and incubated at −20°C for 10 min followed by 20 min at room temperature. Plates were developed with True Blue HRP substrate and imaged on an ELISpot reader. The half maximum inhibitory concentration (IC50) was calculated using normalized counted foci.

### Histology of animal lungs

Mouse lungs were preserved in 10% neutral buffered formalin for 48 h before transferring to 70% ethanol. The tissue sections were then embedded in paraffin blocks and sectioned at 8-μm thickness. Slides were deparaffinized and rehydrated before staining for hematoxylin and eosin for routine immunopathology. IHC was performed on mice lung tissues probed with SARS/SARS-CoV-2 Coronavirus NP Monoclonal Antibody (B46F) (Product No. MA1-7404) at a dilution of 1:100. The antibody showed significant staining in lung tissues of non-immunized, SARS-CoV-2- infected mice when compared to the tissues of the vaccinated group of mice. This method was meant to demonstrate the relative expression of the Nucleocapsid protein between non-immunized Mock and immunized samples. Further CD8^+^ T- cell and CD4^+^ T- cell-specific staining were performed to identify the T- cell infiltration among the immunized and Mock groups.

### Peripheral blood mononuclear cells isolation and T cell stimulation

Peripheral blood mononuclear cells (PBMCs) from COVID-19 patients were isolated from the blood using Ficoll (GE Healthcare) density gradient media and transferred into 96-well plates at a concentration of 2.5 × 10^6^ viable cells per ml in 200 µl (0.5 × 10^6^ cells per well) of RPMI-1640 media (Hyclone) supplemented with 10% (v/v) FBS (HyClone), sodium pyruvate (Lonza), L-glutamine, non-essential amino acids, and antibiotics (Corning). A fraction of the blood was kept separated to perform HLA genotyping of the patients and select only the HLA-A*02:01- and/or DRB1*01: 01-positive individuals. Subsequently, cells were then stimulated with 10 µg/ml of each one of the 22 individual T- cell peptide epitopes (16 CD8^+^ T- cell peptides and 6 CD4^+^ T- cell peptides) and incubated in humidified 5% CO_2_ at 37°C. Post-incubation, cells were stained by flow cytometry analysis or transferred in IFN-γ ELISpot plates. The same isolation protocol was followed for healthy donor (HD) samples obtained in 2018. PBMC samples were kept frozen in liquid nitrogen in 10% FBS in DMSO. Upon thawing, HD PBMCs were stimulated in the same manner for the IFN-γ ELISpot technique.

### ELISpot assay

COVID-19 patients were first screened for their HLA status (DRB1*01:01 positive = 108, HLA-A*02:01 positive = 83, DRB1*01:01 and HLA-A*02:01 positive = 19). PBMC samples from the 108 DRB1*01:01 positive individuals were used to assess the CD4^+^ T-cell response against our SL-CoVs-conserved SARS-CoV-2-derived class-II restricted epitopes by IFN-γ ELISpot. Subsequently, we assessed the CD8^+^ T- cell response against our SL-CoVs conserved SARS-CoV- 2-derived class-I restricted epitopes in the PBMC sample of 83 HLA-A*02:01- positive individuals representing different disease severity categories. Furthermore, to evaluate the immunogenicity of conserved SARS-CoV-2 CD8^+^ and CD4^+^ T- cell epitopes in triple transgenic HLA-A*02:01/HLA-DRB1*01:01-hACE-2 mice, mononuclear cells from lung tissues were collected 14 days post-infection. ELISpot assay was performed as described previously (33, 39).

### Flow cytometry analysis

After 72 h of stimulation with each SARS-CoV-2 class I or class II restricted peptide, PBMCs (0.5 × 10^6^ cells) from 147 patients were stained for the detection of surface markers and subsequently analyzed by flow cytometry. First, the cells were stained with a live/dead fixable dye (Zombie Red dye, 1/800 dilution —BioLegend, San Diego, CA) for 20 min at room temperature, to exclude dying/apoptotic cells. Cells were stained for 45 min at room temperature with five different HLA-A*02*01 restricted tetramers and/or five HLA-DRB1*01:01 restricted tetramers (PE labeled) specific toward the SARS-CoV-2 CD8^+^ T- cell epitopes Orf1ab_2210–2218_, Orf1ab_4283–4291_, S_1220–1228_, ORF10_3–11_ and toward the CD4^+^ T- cell epitopes ORF1a_1350–1365_, S_1–13_, M_176–190_, ORF6_12 26_, respectively. We have optimized our tetramer staining according to the instructions published by Dolton et al. (40) As a negative control aiming to assess tetramer staining specificity, we stained HLA-A*02*01-HLA-DRB1*01:01-negative patients with our four tetramers. Subsequently, we used anti-human antibodies for surface marker staining: anti-CD62L, anti-CD69, anti-CD4, anti-CD8, and anti-IFN-g. mAbs against these various cell markers were added to the cells in phosphate-buffered saline (PBS) containing 1% FBS and 0.1% sodium azide (fluorescence-activated cell sorter [FACS] buffer) and left for 30 min at 4°C. At the end of the incubation period, the cells were washed twice with FACS buffer and fixed with 4% paraformaldehyde (PFA, Affymetrix, Santa Clara, CA). A total of ∼200,000 lymphocyte-gated PBMCs (140,000 alive CD45^+^) were acquired by Fortessa X20 (Becton Dickinson, Mountain View, CA) and analyzed using FlowJo software (TreeStar, Ashland, OR).

### Enzyme-linked immunosorbent assay

Serum antibodies specific for epitope peptides and SARS-CoV-2 proteins were detected by ELISA. The 96-well plates (Dynex Technologies, Chantilly, VA) were coated with 0.5 μg peptides and 100 ng S or N protein per well at 4°C overnight, respectively, and then washed three times with PBS and blocked with 3% BSA (in 0.1% PBST) for 2 h at 37°C. After blocking, the plates were incubated with serial dilutions of the sera (100 μl/well, in twofold dilution) for 2 h at 37°C. The bound serum antibodies were detected with HRP-conjugated goat anti-mouse IgG and chromogenic substrate TMB (Thermo Fisher, Waltham, MA). The cut-off for seropositivity was set as the mean value plus three standard deviations (3SD) in HBc-S control sera. The binding of the epitopes to the sera of SARS-CoV-2- infected samples was detected by ELISA using the same procedure; 96-well plates were coated with 0.5 μg peptides, and sera were diluted at 1:50. All ELISA studies were performed at least twice.

### Data and code availability

The human-specific SARS-CoV-2 complete genome sequences were retrieved from the GISAID database, whereas the SARS-CoV-2 sequences for pangolin (*Manis javanica*) and bat (*Rhinolophus affinis*, *Rhinolophus malayanus*) were retrieved from NCBI. Genome sequences of previous strains of SARS-CoV for humans, bats, civet cats, and camels were retrieved from the NCBI GenBank.

## Results

### Highly conserved SARS-CoV-2 epitopes are selectively recognized by CD8^+^ and CD4^+^ T cells from asymptomatic COVID-19 patients irrespective of variants of concern infection

To identify “universal” SARS-CoV-2 epitopes to be included in a multi-epitope pan-coronavirus vaccine, we previously screened the degree of conservancy for human CD8^+^ T cell, CD4^+^ T cell, and B-cell epitopes that span the whole SARS-CoV-2 genome (33). CD8^+^ T- cell epitopes were screened for their conservancy against variants, namely, h-CoV-2/Wuhan (MN908947.3), h-CoV-2/WA/USA2020 (OQ294668.1), h-CoV-2/Alpha(B1.1.7) (OL689430.1), h-CoV-2/Beta(B 1.351) (MZ314998), h-CoV-2/Gamma(P.1) (MZ427312.1), h-CoV-2/Delta(B.1.617.2) (OK091006.1), and h-CoV-2/Omicron(B.1.1.529) (OM570283.1) (33). We observed 100% conservancy in all the SARS-CoV-2 variants of concern for 14 of our 16 predicted CD8^+^ T- cell epitopes (ORF1ab_2210–2218_, ORF1ab_3013–3021_, ORF1ab_4283–4291_, ORF1ab_6749–6757_, ORF6_3–11_, ORF7b_26–34_, ORF10_3–11_, ORF10_5–13_, S_958–966_, S_1000–1008_, S_1220–1228_, E_20–28_, M_52–60_, and M_89–97_) (**Supplementary Figure S1**) and (33). The only exceptions were epitopes E_26–34_ and ORF8a_73–81_, which showed an 88.8% conservancy against Beta (B.1.351) and Alpha (B.1.1.7) variants, respectively (**Supplementary Figure S1**) and (33). All of the six highly immunodominant “universal” CD4^+^ T- cell epitopes (ORF1a_1350–1365_, ORF6_12–26_, ORF8b_1–15_, S_1–13_, M_176–190_, and N_388–403_) that we previously reported (33) remained 100% conserved across all the SARS-CoV-2 VOCs (**Supplementary Figure S2**).

**Figure 1:**
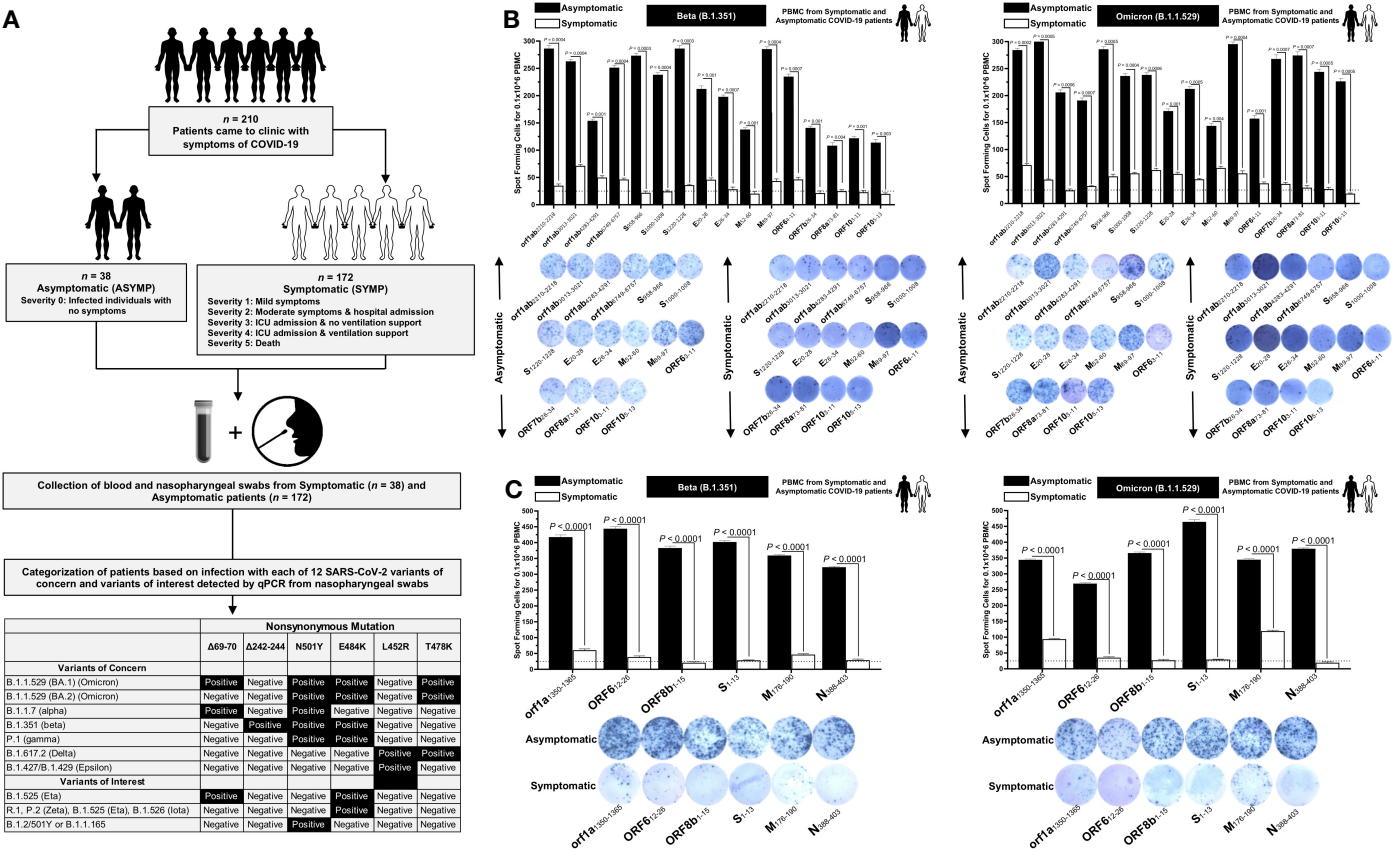
Screening of COVID-19 patients based on SARS-CoV-2 variants and subsequent evaluation of IFN-γ CD8^+^ and CD4^+^ T- cell responses for conserved CD8^+^, and CD4^+^ T-cell epitopes in asymptomatic vs. symptomatic COVID-19 patients: **(A)** experimental plan showing screening process of COVID-19 patients (*n* = 210) into asymptomatic and symptomatic categories based on clinical parameters. Blood and nasopharyngeal swabs were collected from all the subjects, and a qRT-PCR assay was performed. Six novel nonsynonymous mutations (Δ69–70, Δ242–244, N501Y, E484K, L452R, and T478K) were used to identify the haplotypes unique to different SARS-CoV-2 variants of concern (Omicron (B.1.1.529 (BA.1)), Omicron (B.1.1.529 (BA.2)), Alpha (B.1.1.7), Beta (B.1.351), Gamma (P.1), Delta (B.1.617.2), and Epsilon (B.1.427/B.1.429)) and variants of interest (Eta (B.1.525), R.1, Zeta (P.2), Iota (B.1.526), and B.1.2/501Y or B.1.1.165). **(B)** ELISpot images and bar diagrams showing average frequencies of IFN-γ- producing cell spots from immune cells from PBMCs (1 × 10^6^ cells per well) of COVID-19 infected with highly pathogenic SARS-CoV-2 variants of concern Beta (B.1.351) (left panel) and Omicron (B.1.1.529) (right panel). Cells were stimulated for 48 h with 10 mM of 16 immunodominant CD8^+^ T- cell peptides derived from SARS-CoV-2 structural (Spike, Envelope, and Membrane) and non-structural (orf1ab, ORF6, ORF7b, ORF8a, and ORF10) proteins. **(C)** ELISpot images and bar diagrams showing average frequencies of IFN-γ- producing cell spots from immune cells from PBMCs (1 × 10^6^ cells per well) of COVID-19 infected with SARS-CoV-2 variants of concern Alpha (B.1.1.7) (left panel) and Omicron (B.1.1.529) (right panel). Cells were stimulated for 48 h with 10 mM of six immunodominant CD4^+^ T- cell peptides derived from SARS-CoV-2 structural (Spike, Membrane, and Nucleocapsid) and non-structural (ORF1a, ORF6, and ORF8a) proteins. The bar diagrams show the average/mean numbers (± SD) of IFN-γ-spot forming cells (SFCs) after CD8^+^ T- cell peptide-stimulation PBMCs of asymptomatic and symptomatic COVID-19 patients. Dotted lines represent an arbitrary threshold set to evaluate the relative magnitude of the response. A strong response is defined for mean SFCs > 25 per 1 × 10^6^ stimulated PBMCs. Results were considered statistically significant at *p* < 0.05.

Next, we determined whether the highly conserved “universal” CD8^+^ and CD4^+^ T-cell epitopes were differentially recognized by T cells from asymptomatic (ASYMP) versus symptomatic (SYMP) COVID-19 patients. We recruited COVID-19 patients infected with SARS-CoV-2 Beta (B.1.351) and SARS-CoV-2 Omicron (B.1.1.529) spanning 2 years of the COVID-19 pandemic (**Figure 1A**). We compared the magnitude of CD8^+^ and CD4^+^ T- cell responses specific to each of the conserved epitopes from among 38 ASYMP and 172 SYMP COVID-19 patients. Fresh PBMCs were isolated from SYMP and ASYMP COVID-19 patients, on average, within 4 days after reporting their first symptoms. PBMCs were then stimulated *in vitro* for 72 h with each of the 16 CD8^+^ T- cell epitopes or each of the 6 CD4^+^ T cell epitopes. Numbers of responding IFN-γ-producing CD8^+^ and CD4^+^ T cells (quantified in ELISpot assay as the number of IFN-γ-spot forming cells, or “SFCs”) were subsequently determined.

ASYMP COVID-19 patients showed significantly higher frequencies of SARS-CoV-2 epitope-specific IFN-γ-producing CD8^+^ T cells (mean SFCs > 25 per 1 × 10^6^ pulmonary immune cells), irrespective of infection with Beta (*p* < 0.5, **Figure 1B**, left panel) or Omicron (*p* < 0., **Figure 1B**, right panel) variants. In contrast, severely ill or hospitalized symptomatic COVID-19 patients showed significantly lower frequencies of SARS-CoV-2 epitope-specific IFN-γ-producing CD8^+^ T cells (*p* < 0.5, **Figure 1B**, left panel) or Omicron (*p* < 0., **Figure 1B**, right panel) variants. This observation was consistent regardless of whether the CD8^+^ T- cell targeted epitopes were from structural or non-structural SARS-CoV-2 protein antigens, suggesting that strong CD8^+^ T- cell responses specific to selected “universal” SARS-CoV-2 epitopes were commonly associated with better COVID-19 outcomes. In contrast, low SARS-CoV-2-specific CD8^+^ T- cell responses were more commonly associated with severe onset of disease.

Similarly, higher frequencies of functional IFN-γ-producing CD4^+^ T cells ASYMP COVID-19 patients (mean SFCs > 25 per 1 × 10^6^ pulmonary immune cells) were detected, irrespective of infection with Beta (*p* < 0.5, **Figure 1C**, left panel) or Omicron (*p* < 0., **Figure 1C**, right panel) variants, whereas reduced frequencies of IFN-γ-producing CD4^+^ T cells were detected in SYMP COVID-19 patients, irrespective of infection with Beta (*p* < 0.5, **Figure 1C**, left panel) or Omicron (*p* < 0., **Figure 1C**, right panel) variants. This observation was consistent regardless of whether the CD4^+^ T- cell targeted epitopes were from structural or non-structural SARS-CoV-2 protein antigens. Our results suggest that strong CD4^+^ T- cell responses specific to selected “universal” SARS-CoV-2 epitopes were commonly associated with better COVID-19 outcomes. In contrast, low SARS-CoV-2-specific CD4^+^ T- cell responses were more commonly associated with severe disease onset.

Taken together, these results (1) demonstrate an important role of SARS-CoV-2-specific CD4^+^ and CD8^+^ T cells directed against highly conserved structural and non-structural SARS-CoV-2 epitopes in protection from severe COVID-19 symptoms, (2) highlight the potential importance of these highly conserved “asymptomatic” epitopes in mounting protected CD4^+^ and CD8^+^ T cell responses against multiple SARS-CoV-2 VOCs, and (3) support targeting these conserved epitopes with a vaccine.

### A pan-variant SARS-CoV-2 vaccine composed of a mixture of conserved “asymptomatic” CD4^+^ and CD8^+^ T cell epitopes provides robust protection against infection and disease caused by six SARS-CoV-2 variants of concern

We next used a prototype pan-variant SARS-CoV-2 vaccine composed of a mixture of 6 conserved “asymptomatic” CD4^+^ T- cell epitopes and 16 conserved “asymptomatic” CD4^+^ and CD8^+^ T- cell epitopes that span the whole SARS-CoV-2 genome (33). We focused mainly on CD4^+^ and CD8^+^ T- cell epitopes that show immunodominance selectively in SYMP COVID-19 patients infected with various SARS-CoV-2 VOCs.

**Figure 2:**
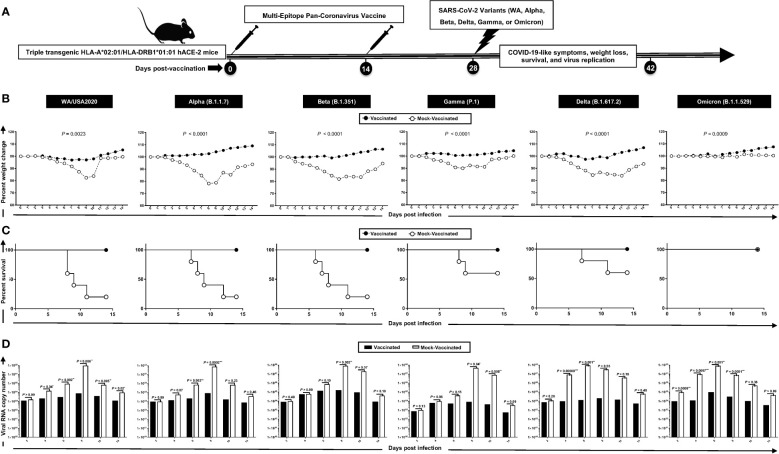
Protection induced against six SARS-CoV-2 variants of concern in triple transgenic HLA-A*02:01/HLA-DRB1*01:01-hACE-2 mice following immunization with a pan-variant SARS-CoV-2 vaccine incorporating conserved human B, CD4^+^, and CD8^+^ T- cell epitopes: **(A)** experimental scheme of vaccination and challenge triple transgenic HLA-A*02:01/HLA-DRB1*01:01-hACE-2 mice. Triple transgenic HLA-A*02:01/HLA-DRB1*01:01-hACE-2 mice (7–8 weeks old, *n* = 60) were immunized subcutaneously on Days 0 and 14 with a multi-epitope pan-variant SARS-CoV-2 vaccine consisting of a pool of conserved B, CD4^+^ T-cell, and CD8^+^ T-cell human epitope peptides. The pool of peptides comprised 25 μg of each of the 16 CD8^+^ T- cell peptides, 6 CD4^+^ T- cell peptides, and 7 B-cell peptides. The final composition of peptides was mixed with 25 μg of CpG and 25 μg of Alum. Mock-vaccinated mice were used as controls (*Mock*). Mice were intranasally challenged with each of the six different SARS-CoV-2 variants of concern (WA/USA2020, Alpha (B.1.1.7), Beta (B.1.351), Gamma (P.1), Delta (B.1.617.2), and Omicron (B.1.1.529)) 14 days following the second immunization. Vaccinated and mock-vaccinated mice were followed 14 days post-challenge for COVID-like symptoms, weight loss, survival, and virus replication. **(B)** Percent weight change recorded daily for 14 days p.i. in vaccinated and mock-vaccinated mice following the challenge with each of the six different SARS-CoV-2 variants. **(C)** Kaplan–Meir survival plots for vaccinated and mock-vaccinated mice following the challenge with each of the six different SARS-CoV-2 variants. **(D)** Virus replication in vaccinated and mock-vaccinated mice following the challenge with each of the six different SARS-CoV-2 variants detected in throat swabs on Days 2, 4, 6, 8, 10, and 14, The indicated *p*-values are calculated using the unpaired *t*-test, comparing results obtained in vaccinated VERSUS mock-vaccinated mice. Statistical significance was obtained when *: P < 0.05, **: P < 0.01, ***:P < 0.001, ****:P < 0.0001.

A pool of peptides comprising 25 μg each of 16 CD8^+^ T- cell peptides (ORF1ab_2210–2218_, ORF1ab_3013–3021_, ORF1ab_4283–4291_, ORF1ab_6749–6757_, ORF6_3–11_, ORF7b_26–34_, ORF8a_73–81_, ORF10_3–11_, ORF10_5–13_, S_958–966_, S_1000–1008_, S_1220–1228_, E_20–28_, E_26–34_, M_52–60_, and M_89–97_), 6 CD4^+^ T- cell epitopes (ORF1a_1350–1365_, ORF6_12–26_, ORF8b_1–15_, S_1–13_, M_176–190_, and N_388–403_), and 7 B-cell peptides selected from the Spike protein were mixed with cpG1826 adjuvant and administered subcutaneously on Day 0 and Day 14 to 7–8- week- old triple transgenic HLA-A*02:01/HLA-DR hACE-2 mice (*n* = 30). The remaining group (mock-immunized) received vehicle alone (*n* = 30) (**Figure 2A**). On day 28, 14 days after the second immunization, mice were divided into six groups and intranasally infected with 1 × 10^5^ pfu of SARS-CoV-2 (USA-WA1/2020) (*n* = 10), 6 × 10^3^ pfu of SARS-CoV-2-Alpha (B.1.1.7) (*n* = 10), 6 × 10^3^ pfu of SARS-CoV-2-Beta (B.1.351) (*n* = 10), 5 × 10^2^ pfu of SARS-CoV-2-Gamma (P.1) (*n* = 10), 8 × 10^3^ pfu of SARS-CoV-2-Delta (B.1.617.2) (*n* = 10), and 6.9 × 10^4^ pfu of SARS-CoV-2-Omicron (B.1.1.529) (*n* = 10) (**Figure 2A**).

Mice that received the pan-variant SARS-CoV-2 vaccine showed significant protection from weight loss (**Figure 2B**) and death (**Figure 2C**) following infection with each of the six SARS-CoV-2 variants of concern. In contrast, mock-immunized mice showed substantial mortality against infection with WA/USA2020 (60%), Alpha (B.1.1.7) (80%), Beta (B.1.351) (80%), Gamma (P.1) (40%), and Delta (B.1.617.2) (40%) (**Figure 2C**). Mortality was not observed for mock-immunized mice infected with the SARS-CoV-2 Omicron (B.1.1.529) variant (**Figure 2C**).

Throat swabs were collected from the vaccinated and mock-vaccinated groups of mice on days 2, 4, 6, 8, 10, and 14 post-infection (p.i.) and were processed to detect the viral RNA copy number by qRT-PCR (**Figure 2D**). Compared to the viral RNA copy number detected from the mock-vaccinated group of mice, we detected a statistically significant decrease in the viral RNA copy number among vaccinated groups of mice on day 4 p.i. for SARS-CoV-2 WA/USA2020 (*p* = 0.04), Delta (B.1.617.2) (*p* = 0.00009), and Omicron (B.1.1.529) (*p* = 0.007); on day 6 p.i. for SARS-CoV-2 WA/USA2020 (*p* = 0.002), Alpha (B.1.1.7) (*p* = 0.002), Delta (B.1.617.2) (*p* = 0.001), and Omicron (B.1.1.529) (*p* = 0.001); on day 8 p.i. for SARS-CoV-2 WA/USA2020 (*p* = 0.006), Alpha (B.1.1.7) (*p* = 0.0002), Beta (B.1.351) (*p* = 0.002), Gamma (P.1) (*p* = 0.04), and Omicron (B.1.1.529) (*p* = 0.0001); on day 10 p.i. for SARS-CoV-2 WA/USA2020 (*p* = 0.005), Gamma (P.1) (*p* = 0.008); and on day 14 p.i. for SARS-CoV-2 WA/USA2020 (*p* = 0.02) (**Figure 2D**). These results demonstrate that the pan-variant SARS-CoV-2 vaccine conferred significant protection from virus replication against SARS-CoV-2 variants and supports the hypothesis that a broad anti-viral effect following immunization with asymptomatic B and CD4^+^ and CD8^+^ T- cell epitopes carefully selected as being highly conserved from multiple SARS-CoV-2 variants.

### Immunization with the pan-variant SARS-CoV-2 vaccine bearing conserved epitopes reduced COVID-19-related lung pathology and virus replication associated with increased infiltration of CD8^+^ and CD4^+^ T cells in the lungs

**Figure 3:**
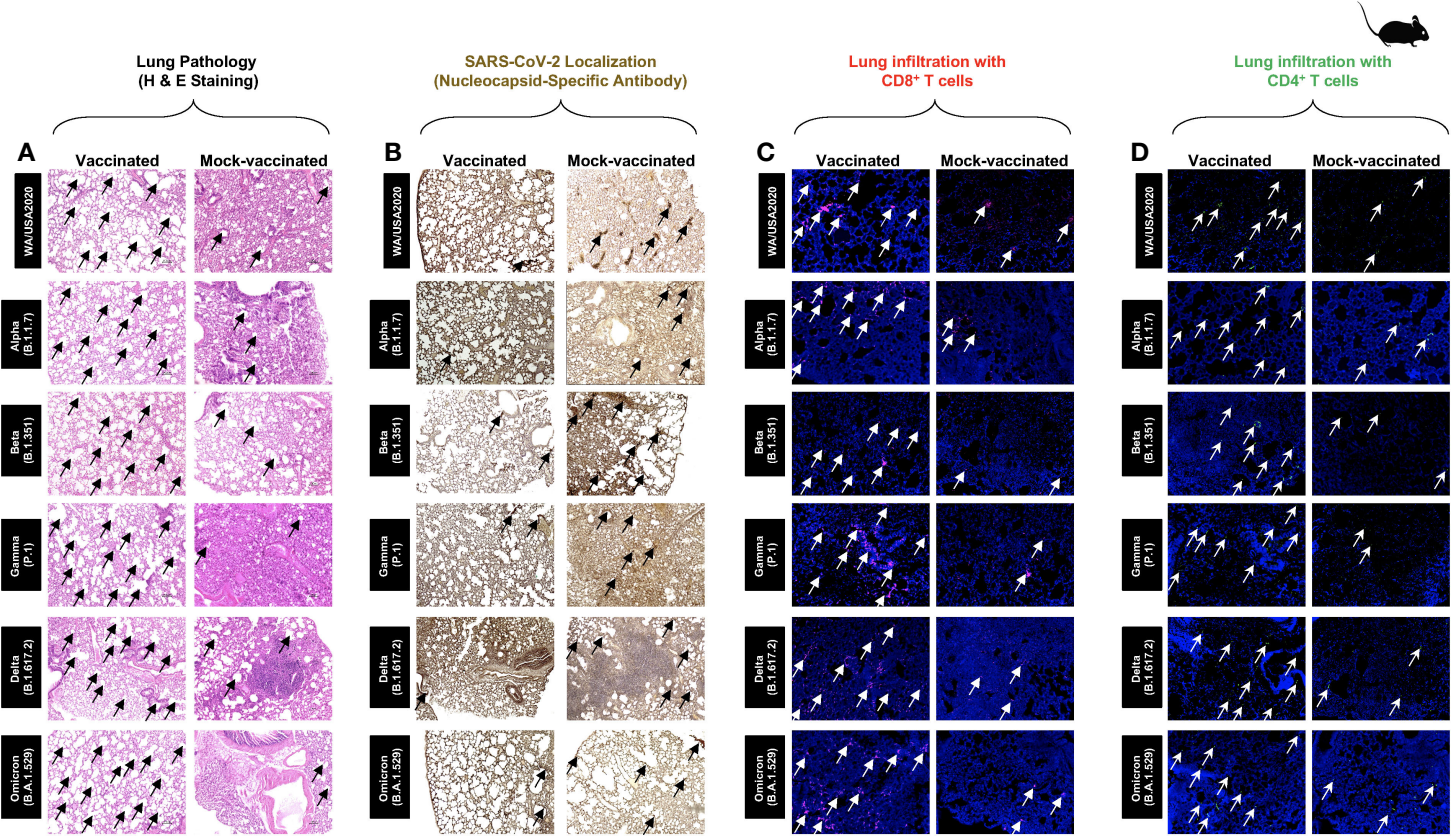
Histopathology and immunohistochemistry of the lungs from in triple transgenic HLA-A*02:01/HLA-DRB1*01:01-hACE-2 mice vaccinated and mock-vaccinated mice. **(A)** Representative images of hematoxylin and eosin (H & E) staining of the lungs harvested on day 14 p.i. from vaccinated (left panels) and mock-vaccinated (right panels) mice. **(B)** Representative immunohistochemistry (IHC) sections of the lungs were harvested on Day 14 p.i. from vaccinated (left panels) and mock-vaccinated (right panels) mice and stained with SARS-CoV-2 Nucleocapsid antibody. Black arrows point to the antibody staining. Fluorescence microscopy images showing infiltration of CD8^+^ T cells **(C)** and of CD4^+^ T cells **(D)** in the lungs from vaccinated (left panels) and mock-vaccinated (right panels) mice. Lung sections were co-stained using DAPI (*blue*) and mAb specific to CD8^+^ T cells (pink) (magnification, ×20). The white arrows point to CD8^+^ and CD4^+^ T cells infiltrating the infected lungs.

Hematoxylin and eosin staining of lung sections at day 14 p.i. showed a significant reduction in COVID-19-related lung pathology in the mice immunized with conserved pan-variant SARS-CoV-2 vaccine compared to mock-vaccinated mice (**Figure 3A**). This reduction in lung pathology was observed for all six SARS-CoV-2 variants: USA-WA1/2020, Alpha (B.1.1.7), Beta (B.1.351), Gamma (P.1), Delta (B.1.617.2), and Omicron (B.1.1.529) (**Figure 3A**). We further performed SARS-CoV-2 Nucleocapsid Antibody- Based Immunohistochemistry (IHC) staining on lung tissues obtained from vaccinated and mock-vaccinated groups of mice infected with SARS-CoV-2 variants. We detected significantly lower antibody staining in the lung tissues of the vaccinated compared mock-vaccinated group of mice following infection with each of the six SARS-CoV-2 variants of concern. This indicated higher expression of the target viral proteins in the lungs of the mock-vaccinated compared to the vaccinated group of mice (**Figure 3B**). Furthermore, IHC staining was performed to compare the infiltration CD8^+^ and CD4^+^ T cells into lung tissues of vaccinated and mock-vaccinated mice infected with various SARS-CoV-2 variants. We observed a significant increase in the infiltration of both CD8^+^ T cells (**Figure 3C**) and CD4^+^ T cells (**Figure 3D**) in the lungs of vaccinated mice compared to mock-vaccinated mice 14 days following infection with each of the six variants.

Taken together, these results indicate that immunization with the pan-variant SARS-CoV-2 vaccine bearing conserved epitopes induced cross-protective CD8^+^ and CD4^+^ T cells that infiltrated the lungs, faciltated clearance of virus, and reduced COVID-19-related lung pathology following infection with various multiple SARS-CoV-2 variants.

### Increased frequencies of lung-resident functional CD8^+^ and CD4^+^ T_EM_ and T_RM_ cells induced by the pan-variant SARS-CoV-2 vaccine are associated with protection against multiple SARS-CoV-2 variants

**Figure 4:**
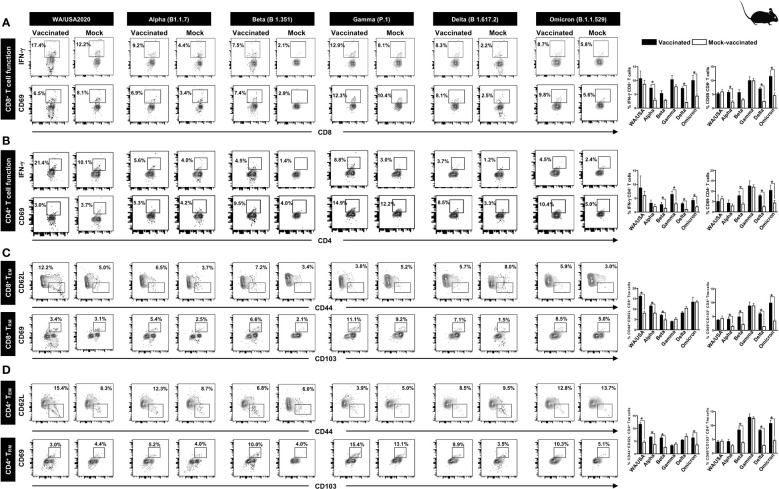
The effect of pan-coronavirus immunization on CD8^+^ and CD4^+^ T- cell function and memory response: FACS plots and bar graphs showing the **(A)** expression of CD8^+^ T- cell function markers, **(B)** CD4^+^ T- cell function associated markers, **(C)** CD8^+^ T effector memory response (CD44^+^CD62L^-^), and CD8^+^ T resident memory (CD103^+^CD69^+^) response, and **(D)** CD4^+^ T effector memory response (CD44^+^CD62L ^−^), and CD4^+^ resident memory (CD103^+^CD69^+^) response in the lung of vaccinated and mock-vaccinated groups of mice infected with multiple SARS-CoV-2 variants. Mononuclear cells from lung tissue were collected 14 days post-infection. Bars represent means ± SEM. Data were analyzed by Student’s *t*-test. Results were considered statistically significant at *p* < 0.05.

To determine whether increased frequencies of lung-resident functional CD8^+^ and CD4^+^ T cells induced by the pan-variant SARS-CoV-2 vaccine are associated with protection against multiple SARS-CoV-2 variants, we used flow cytometry to compared the frequencies of IFN-γ CD8^+^ T cells and CD69 CD8^+^ T cells (**Figure 4A**), IFN-γ CD4^+^ T cells and CD69 CD4^+^ T cells (**Figure 4B**) in cell suspensions from the lungs of vaccinated versus mock-vaccinated groups of mice.

Relatively higher frequencies of IFN-γ CD8^+^ T cells were detected in the lungs of protected mice that received the pan-variant SARS-CoV-2 vaccine compared to non-protected mock-vaccinated mice following infections with various SARS-CoV-2 variants: USA-WA1/2020 (Vaccinated = 17.4% vs. Mock = 12.2%, *p* = 0.5178), Alpha (B.1.1.7) (Vaccinated = 9.2% vs. Mock = 4.4%, *p* = 0.0076), Beta (B.1.351) (Vaccinated = 7.5% vs. Mock = 2.1%, *p* = 0.05), Gamma (P.1) (Vaccinated = 12.9% vs. Mock = 8.1%, *p* = 0.14), Delta (B.1.617.2) (Vaccinated = 8.3% vs. Mock = 2.23%, *p* < 0.0001), and Omicron (B.1.1.529) (Vaccinated = 8.7% vs. Mock = 5.8%, *p* = 0.02) (**Figure 4A**, top row). Similarly, increased frequencies for CD8^+^CD69^+^ T cells were detected in the lungs of protected mice that received the pan-variant SARS-CoV-2 vaccine compared to non-protected mock-vaccinated mice following infections with various SARS-CoV-2 variants: Alpha (B.1.1.7) (Vaccinated = 6.9% vs. Mock = 3.4%, *p* = 0.0033), Beta (B.1.351) (Vaccinated = 7.4% vs. Mock = 2.9%, *p* = 0.05), Gamma (P.1) (Vaccinated = 12.3% vs. Mock = 10.4%, *p* = 0.95), Delta (B.1.617.2) (Vaccinated = 8.1% vs. Mock = 2.5%, *p* < 0.0001), and Omicron (B.1.1.529) (Vaccinated = 9.8% vs. Mock = 5.6%, *p* = 0.01) (**Figure 4A**, bottom row).

Moreover, higher frequencies of IFN-γ CD4^+^ T cells were detected in the lungs of protected mice that received the pan-variant SARS-CoV-2 vaccine compared to non-protected mock-vaccinated mice following infections with some of the SARS-CoV-2 variants: USA-WA1/2020 (Vaccinated = 21.4% vs. Mock = 10.1%, *p* = 0.5696), Alpha (B.1.1.7) (Vaccinated = 5.6% vs. Mock = 4%, *p* = 0.35), Beta (B.1.351) (Vaccinated = 4.5% vs. Mock = 1.4%, *p* = 0.01), Gamma (P.1) (Vaccinated = 8.8% vs. Mock = 3%, *p* = 0.02), Delta (B.1.617.2) (Vaccinated = 3.7% vs. Mock = 1.2%, *p* = 0.0002), and Omicron (B.1.1.529) (Vaccinated = 4.5% vs. Mock = 2.4%, *p* = 0.01) (**Figure 4B**, top row). Similarly, increased frequencies for CD4^+^CD69^+^ T cells were detected in the lungs of protected mice that received the pan-variant SARS-CoV-2 vaccine compared to non-protected mock-vaccinated mice following infections with various SARS-CoV-2 variants: Alpha (B.1.1.7) (Vaccinated = 5.3% vs. Mock = 4.2%, *p* = 0.1748), Beta (B.1.351) (Vaccinated = 9.5% vs. Mock = 4%, *p* = 0.009), Gamma (P.1) (Vaccinated = 14.9% vs. Mock = 12.2%, *p* = 0.7155), Delta (B.1.617.2) (Vaccinated = 8.5% vs. Mock = 3.3%, *p* < 0.0001), and Omicron (B.1.1.529) (Vaccinated = 10.4% vs. Mock = 5%, *p* = 0.003) (**Figure 4B**, bottom row).

FACS-based immunophenotyping, confirmed higher frequencies of the memory CD8^+^ T_EM_ (CD44^+^CD62L ^−^) cell subset in immunized mice with a pool of pan- coronavirus peptides and subjected to infection against USA-WA1/2020 (Vaccinated = 12.2% vs. Mock = 5%, *p* < 0.0001), Alpha (B.1.1.7) (Vaccinated = 6.5% vs. Mock = 3.7%, *p* = 0.0017), Beta (B.1.351) (Vaccinated = 7.2% vs. Mock = 3.4%, *p* = 0.0253), and Omicron (B.1.1.529) (Vaccinated = 5.9% vs. Mock = 3%, *p* = 0.9765) (**Figure 4C**). Similarly, when the frequencies of the memory CD8^+^ T_RM_ (CD69^+^CD103^+^) cell subset was evaluated, we found generally higher CD8^+^ T_RM_ cell subset frequencies for immunized mice infected with USA-WA1/2020 (Vaccinated = 3.4% vs. Mock = 3.1%, *p* = 0.4004), Alpha (B.1.1.7) (Vaccinated = 5.4% vs. Mock = 2.5%, *p* = 0.0160), Beta (B.1.351) (Vaccinated = 6.6% vs. Mock = 2.1%, *p* = 0.0420), Gamma (P.1) (Vaccinated = 11.1% vs. Mock = 9.2%, *p* = 0.9961), Delta (B.1.617.2) (Vaccinated = 7.1% vs. Mock = 1.5%, *p* < 0.0001), and Omicron (B.1.1.529) (Vaccinated = 8.5% vs. Mock = 5%, *p* = 0.0139) (**Figure 4C**).

Moreover, in context to memory CD4^+^ T_EM_ (CD44^+^CD62L ^−^) cell subset, relatively higher frequencies were observed for immunized mice subjected to infection with SARS-CoV-2 variants USA-WA1/2020 (Vaccinated = 15.4% vs. Mock = 8.3%, *p* = 0.0001), Alpha (B.1.1.7) (Vaccinated = 12.3% vs. Mock = 8.7%, *p* < 0.0001), and Beta (B.1.351) (Vaccinated = 6.8% vs. Mock = 6%, *p* < 0.0004) (**Figure 4D**). Generally higher frequencies of the CD4^+^ T_RM_ (CD69^+^CD103^+^) cell subset were found in immunized mice infected with SARS-CoV-2 variants Alpha (B.1.1.7) (Vaccinated = 5.2% vs. Mock = 4%, *p* = 0.0828), Beta (B.1.351) (Vaccinated = 10% vs. Mock = 4%, *p* = 0.005), Gamma (P.1) (Vaccinated = 15.4% vs. Mock = 13.1%, *p* = 0.7860), Delta (B.1.617.2) (Vaccinated = 8.9% vs. Mock = 3.5%, *p* < 0.0001), and Omicron (B.1.1.529) (Vaccinated = 10.3% vs. Mock = 5.1%, *p* = 0.0021) (**Figure 4D**).

These findings suggest that immunization with the pan-variant SARS-CoV-2 vaccine bearing conserved epitopes induced high frequencies of functional CD8^+^ and CD4^+^ T_EM_ and T_RM_ cells that infiltrated the lungs, and were associated with a significant decrease in virus replication and reduction in COVID-19-related lung pathology following infection with various multiple SARS-CoV-2 variants.

### Increased SARS-CoV-2 epitopes-specific IFN-γ-producing CD8^+^ T cells in the lungs of vaccinated mice in comparison to mock-vaccinated mice

**Figure 5:**
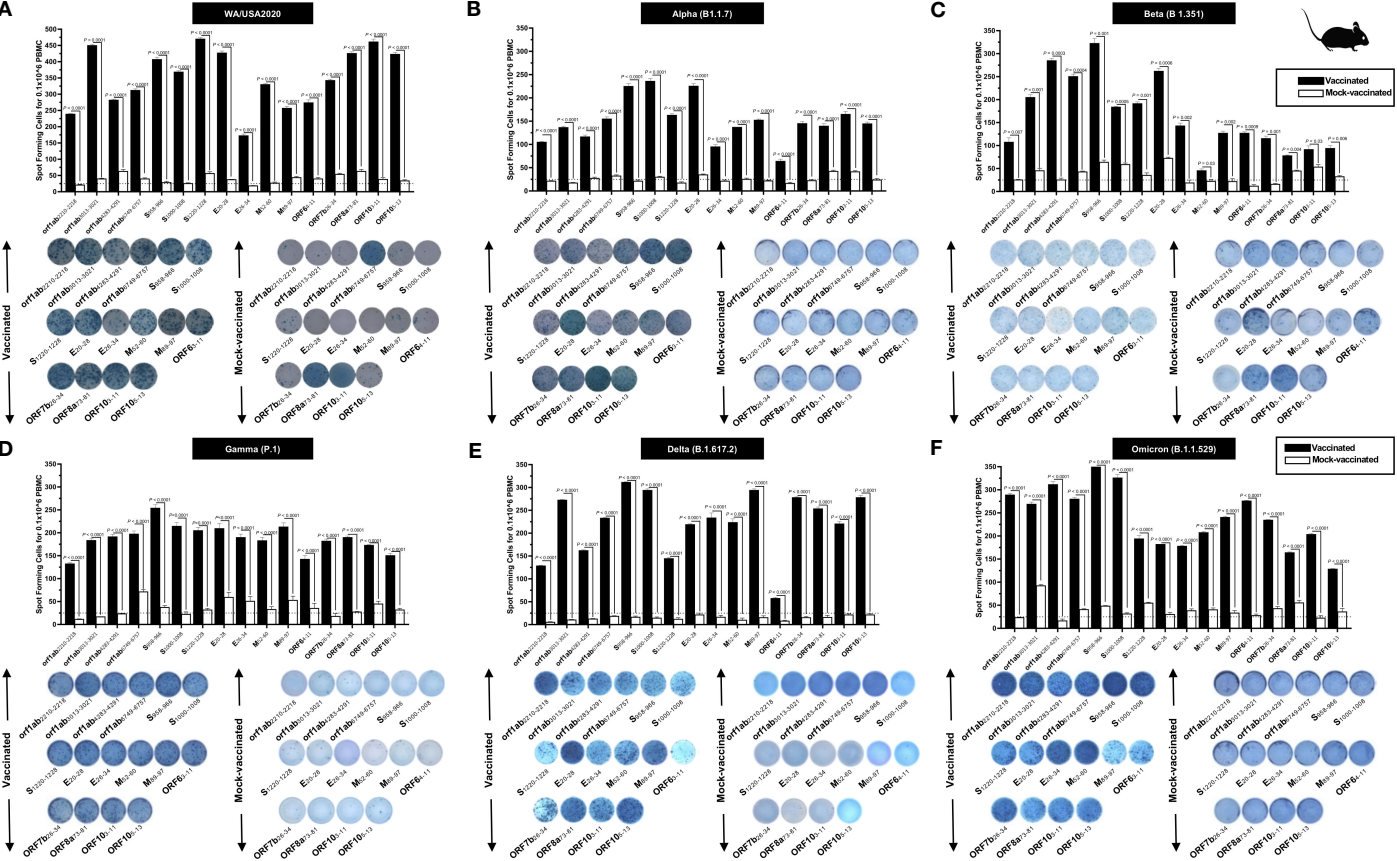
Immunogenicity of conserved SARS-CoV-2 CD8^+^ T- cell epitopes in triple transgenic HLA-A*02:01/HLA-DRB1*01:01-hACE-2 mice: ELISpot images and bar diagrams showing average frequencies of IFN-γ- producing cell spots from mononuclear cells from lung tissue (1 × 10^6^ cells per well) of vaccinated and mock-vaccinated mice challenged with **(A)** WA/USA2020, **(B)** Alpha (B.1.1.7), **(C)** Beta (B.1.351), **(D)** Gamma (P.1), **(E)** Delta (B.1.617.2), and **(F)** Omicron (B.1.1.529). Mononuclear cells from lung tissue were collected 14 days post- infection. The cells were stimulated for 48 h with 10 mM of 16 immunodominant CD8^+^ T- cell peptides. The bar diagrams show the average/mean numbers (± SD) of IFN-γ-spot forming cells (SFCs) after CD8^+^ T- cell peptide stimulation in lung tissues of vaccinated and mock-vaccinated mice. Dotted lines represent an arbitrary threshold set to evaluate the relative magnitude of the response. A strong response is defined for mean SFCs > 25 per 1 × 10^6^ stimulated PBMCs. Results were considered statistically significant at *p* < 0.05.

To determine whether the functional lung-resident CD8^+^ T cells are specific to SARS-CoV-2, we stimulated lung-cell suspension from vaccinated and mock-vaccinated mice with each of the 14 “universal” human CD8^+^ T- cell epitopes (ORF1ab_2210–2218_, ORF1ab_3013–3021_, ORF1ab_4283–4291_, ORF1ab_6749–6757_, ORF6_3–11_, ORF7b_26–34_, ORF10_3–11_, ORF10_5–13_, S_958–966_, S_1000–1008_, S_1220–1228_, E_20–28_, M_52–60_, and M_89–97_) and quantified the number of IFN-γ-producing CD8^+^ T cells using ELISpot, as detailed in *Materials and methods* (**Figure 5**). To determine whether cross-reactive IFN-γ-producing CD8^+^ T- cell responses will be detected regardless of SARS-CoC-2 variant, the number IFN-γ-producing CD8^+^ T cells were determined in the lung tissues of vaccinated and mock-vaccinated mice after challenge with each of six different SARS-CoV-2 variants of concern.

Overall, a significant increase in the number of IFN-γ-producing CD8^+^ T cells was detected in the lungs of protected mice that received the pan-variant SARS-CoV-2 vaccine compared to non-protected mock-vaccinated mice (mean SFCs > 25 per 0.5 × 10^6^ pulmonary immune cells), irrespective of the SARS-CoV-2 variants of concern: WA/USA2020 (**Figure 5A**), Alpha (B.1.1.7) (**Figure 5B**), Beta (B.1.351) (**Figure 5C**), Gamma (P.1) (**Figure 5D**), Delta (B.1.617.2) (**Figure 5E**), or Omicron (B.1.1.529) (**Figure 5F**). All the comparisons among vaccinated and mock-vaccinated groups of mice, irrespective of SARS-CoV-2 variants of concern, were found to be statistically significant regardless of whether CD8^+^ T-cell targeted epitopes were from structural (Spike, Envelope, and Membrane) or non-structural (ORF1ab, ORF6, ORF7b, and ORF10) SARS-CoV-2 protein antigens (*p* < 0.5).

Taken together, these results (1) confirm that immunization with the pan-variant SARS-CoV-2 vaccine bearing conserved epitopes induced high frequencies of functional CD8^+^ T cells that infiltrated the lungs and were associated with cross-protection against multiple SARS-CoV-2 variants; (2) demonstrate that increased SARS-CoV-2 epitope-specific IFN-γ-producing CD8^+^ T cells in the lungs of vaccinated triple transgenic HLA-A*02:01/HLA-DRB1*01:01-hACE-2 mice were associated with protection from multiple variants of concern. In contrast, low frequencies of lung-resident SARS-CoV-2-specific IFN-γ-producing CD8^+^ T cells were associated with severe disease onset in mock-vaccinated triple transgenic HLA-A*02:01/HLA-DRB1*01:01-hACE-2 mice. In this report, we suggest an important role for functional lung-resident SARS-CoV-2-specific CD8^+^ T cells specific to highly conserved “universal” epitopes from structural and non-structural antigens in cross-protection against SARS-CoV-2 VOCs.

### Increased SARS-CoV-2 epitopes-specific IFN-γ-producing CD4^+^ T cells in the lungs of vaccinated mice in comparison to mock-vaccinated mice

**Figure 6:**
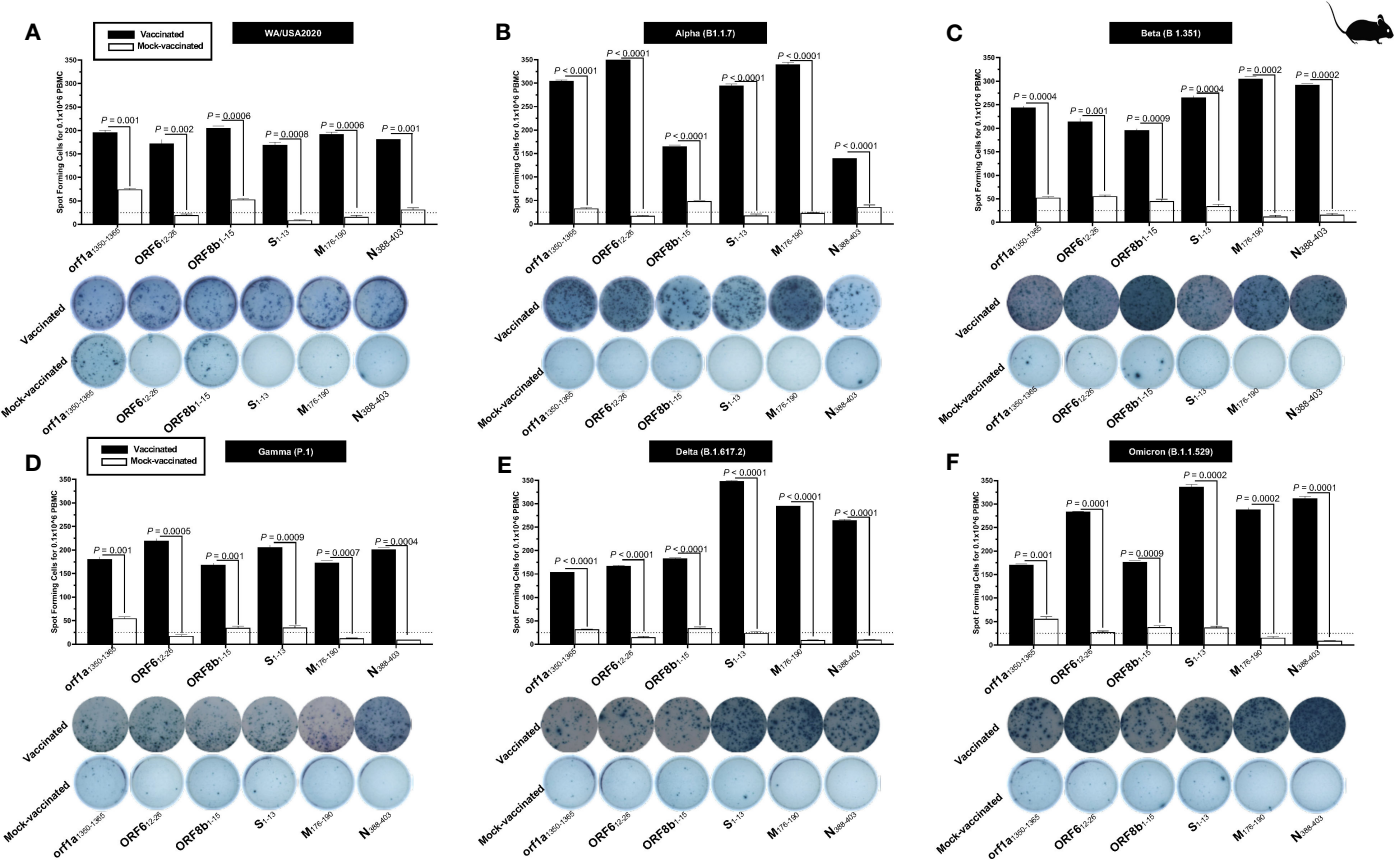
The magnitude of the IFN-γ CD4^+^ T- cell responses for six conserved SARS-CoV-2 CD4^+^ T- cell epitopes in triple transgenic HLA-A*02:01/HLA-DRB1*01:01-hACE-2 mice: ELISpot images and bar diagrams showing average frequencies of IFN-γ producing cell spots from mononuclear cells from lung tissue (1 × 10^6^ cells per well) of vaccinated and mock-vaccinated mice challenged with **(A)** WA/USA2020, **(B)** Alpha (B.1.1.7), **(C)** Beta (B.1.351), **(D)** Gamma (P.1), **(E)** Delta (B.1.617.2), and **(F)** Omicron (B.1.1.529). Mononuclear cells from lung tissue were collected 14 days post-infection. Cells were stimulated for 48 h with 10 mM of six immunodominant CD4^+^ T- cell peptides derived from SARS-CoV-2 structural (Spike, Envelope, and Membrane) and non-structural (orf1ab, ORF6, ORF7b, ORF8a, and ORF10) proteins. The bar diagrams show the average/mean numbers (± SD) of IFN-γ-spot forming cells (SFCs) after CD8^+^ T- cell peptide stimulation in lung tissues of vaccinated and mock-vaccinated mice. The dotted lines represent an arbitrary threshold set to evaluate the relative magnitude of the response. A strong response is defined for mean SFCs > 25 per 1 × 10^6^ stimulated PBMCs. Results were considered statistically significant at *p* ≤ 0.05.

We stimulated lung-cell suspension from vaccinated and mock-vaccinated groups of mice with each of the six “universal” human CD4^+^ T- cell epitopes (ORF1a_1350–1365_, ORF6_12–26_, ORF8b_1–15_, S_1–13_, M_176–190_, and N_388–403_) and quantified the number of IFN-γ-producing CD4^+^ T cells using ELISpot, to determine whether the functional lung-resident CD4^+^ T cells are specific to SARS-CoV-2 (**Figure 6**).

Overall, we detected a significant increase in the number of IFN-γ-producing CD4^+^ T cells in the lungs of protected mice that received the pan-variant SARS-CoV-2 vaccine compared to non-protected mock-vaccinated mice (mean SFCs > 25 per 0.5 × 10^6^ pulmonary immune cells), irrespective of the SARS-CoV-2 VOCs: WA/USA2020 (**Figure 6A**), Alpha (B.1.1.7) (**Figure 6B**), Beta (B.1.351) (**Figure 6C**), Gamma (P.1) (**Figure 6D**), Delta (B.1.617.2) (**Figure 6E**), or Omicron (B.1.1.529) (**Figure 6F**). All the comparisons among vaccinated and mock-vaccinated groups of mice, irrespective of SARS-CoV-2 VOCs, were statistically significant regardless of whether CD4^+^ T-cell targeted epitopes were from structural or non-structural SARS-CoV-2 protein antigens (*p* < 0.5).

Taken together, our findings demonstrate that increased SARS-CoV-2 epitopes-specific IFN-γ-producing CD4^+^ T cells in the lungs of vaccinated triple transgenic HLA-A*02:01/HLA-DRB1*01:01-hACE-2 mice were associated with protection against multiple variants of concern. In contrast, low frequencies of lung-resident SARS-CoV-2-specific IFN-γ-producing CD4^+^ T cells were associated with severe disease onset in mock-vaccinated triple transgenic HLA-A*02:01/HLA-DRB1*01:01-hACE-2 mice. The findings suggest an important role of functional lung-resident SARS-CoV-2-specific CD4^+^ T cells specific to highly conserved “universal” epitopes from structural and non-structural antigens in cross-protection against SARS-CoV-2 VOCs.

### Universal B- cell epitopes from SARS-CoV-2 Spike protein showed a high degree of immunogenicity across SARS-CoV-2 variants based on antibody response in COVID-19 patients and triple transgenic HLA-A*02:01/HLA-DRB1*01:01-hACE-2

We next determined whether the antibody responses were associated with protection, since the prototype pan-variant SARS-CoV-2 vaccine used herein also contains nine conserved B- cell epitopes selected from the Spike glycoprotein of SARS-CoV-2. The nine B-cell epitopes were screened for their conservancy against variants, namely, h-CoV-2/Wuhan (MN908947.3), h-CoV-2/WA/USA2020 (OQ294668.1), h-CoV-2/Alpha (B1.1.7) (OL689430.1), h-CoV-2/Beta (B 1.351) (MZ314998), h-CoV-2/Gamma (P.1) (MZ427312.1), h-CoV-2/Delta (B.1.617.2) (OK091006.1), and h-CoV-2/Omicron (B.1.1.529) (OM570283.1). We observed 100% conservancy in three of our earlier predicted B- cell epitopes, namely, S_287–317_, S_524–558_, and S_565–598_ (**Supplementary Figure S3**).

The antibody titer specific to each of the nine “universal” B-cell epitopes was determined by ELISA in COVID-19 patients infected with multiple SARS-CoV-2 variants of concern (**Supplementary Figure S4**, left panel) and in vaccinated and mock-vaccinated triple transgenic HLA-A*02:01/HLA-DRB1*01:01-hACE-2 mice challenged with same SARS-CoV-2 VOCs (**Supplementary Figure S4**, right panel). The peptide binding IgG level was significantly higher for all nine “universal” B cell epitopes in COVID-19 patients (**Supplementary Figure S4**, left panel) and in vaccinated triple transgenic mice (**Supplementary Figure S4**, right panel), irrespective of SARS-CoV-2 variant. Reduced peptide binding IgG level was observed for severely ill COVID-19 patients (**Supplementary Figure S4**, left panel) and in mock-vaccinated triple transgenic HLA-A*02:01/HLA-DRB1*01:01-hACE-2 mice (**Supplementary Figure S4**, right panel).

Altogether, these results indicate that immunization with the pan-variant SARS-CoV-2 vaccine bearing conserved “universal” B- and T- cell epitope induced cross-protective antibodies, CD8^+^ and CD4^+^ T cells that infiltrated the lungs, facilitate virus clearance, and reduced COVID-19-related lung pathology following infection with various multiple SARS-CoV-2 VOCs.

## Discussion

COVID-19 remains a serious threat with continued high rates of morbidity and mortality worldwide. The ongoing emergence of SARS-CoV-2 variants and sub-variants of concern, including the recent heavily mutated and highly transmissible Omicron sub-variants, has led to vaccine breakthroughs that have contributed to prolonging the COVID-19 pandemic. Current Spike-based COVID-19 vaccines have made a substantial impact on the severity of the pandemic. Neutralizing antibody titers induced by current Spike-based vaccines are less effective against recent variants and sub-variants (41, 42), pointing to the urgent need to develop a next-generation B- and T-cell-based pan-variant SAS-CoV-2 vaccine–coronavirus vaccine that would be based not only on Spike protein but also on less-mutated non-Spike structural and non-structural antigens and epitopes. Such a universal CoV vaccine could induce broader and more durable protective immunity against infections and diseases caused by multiple emerging SARS-CoV-2 variants and sub-variants.

Much of the data on the efficacy of the current modified messenger RNA (mRNA) vaccines has shown that these vaccines elicited lower levels of neutralizing antibodies against newer SARS-CoV-2 variants than against the older variants (41). In the present report, we have identified “universal” CD8^+^ and CD4^+^ T- and B-cell epitopes conserved among all known SARS-CoV-2 variants, previous SARS and MERS coronavirus strains, and strains specific to different species that were reported to be hosts for SARS/MERS (bat, civet cat, pangolin, and camel). We used a combination of these highly conserved CD8+ and CD4+ T- and B- cell epitopes to design a multi-epitope pan-variant SARS-CoV-2 vaccine. The T- cell epitopes that constitute this pan-variant SARS-CoV-2 vaccine represent structural (Spike, Envelope, Membrane, and Nucleocapsid) and non-structural (orf1ab, ORF6, ORF7, ORF8, and ORF10) proteins.

We demonstrated that immunization of triple transgenic h-ACE-2-HLA-A2/DR mice with a pool of “universal” CD8^+^ T-cell, CD4^+^ T- cell, and B- cell peptides conferred protection against Washington, Alpha (B.1.1.7), Beta (B.1.351), Gamma (P.1), Delta (B.1.617.2), and Omicron (B.1.1.529) variants of SARS-CoV-2. The pan-variant SARS-CoV-2 vaccine was found to be safe, as no local or systemic side effects were observed in the vaccinated mice. Moreover, we found that the protection correlated with high frequencies of IFN-γ CD4^+^ T cells, CD69 CD4^+^ T cells, IFN-γ CD8+ T cells, and CD69 CD8+ T cells infuriating the lungs. We also found higher frequencies for the CD8^+^ T_EM_ (CD44^+^CD62L ^−^) cell population in the lungs of protected mice. High levels of peptide-specific IgG were also detected in protected animals, suggesting the contribution of Spike-specific antibodies in protection. A marked difference in the level of neutralizing viral titer was also observed between the vaccinated and mock-vaccinated groups of mice for all the studied variants. We observed no mortality in the vaccinated mice, irrespective of the SARS-CoV-2 variant. In contrast, high mortality was observed in the mock-vaccinated mice when challenged with six SARS-CoV-2 variants. The weight loss and survival shown in this study agree with previous reports in the context to Omicron B.1.1.529 infection in the mouse models (43). Limited weight loss and less virus replication were reported for Omicron B.1.1.529 in 129, C57BL/6, BALB/c, and K18-hACE2 transgenic mice models in comparison to other SARS-CoV-2 variants of concern, such as Alpha (B.1.1.7), Beta (B.1.351), and Delta (B.1.617.2) (43). Moreover, like mouse models, in both wild-type and hACE2 hamsters, a similar trend of milder lung infection, clinical disease, and pathology was observed using the B.1.1.519 variant of Omicron (43). Liu et al. have compared T- cell responses in subjects who received either mRNA or adenovirus-vectored vaccine (44). Both vaccines induced substantial spike-specific IFN-γ-producing CD8^+^ and CD4^+^ T cells, which showed similar reactivity against Wuhan, Delta, and Omicron variants (44). In addition, their findings showed that central and effector memory T- cell subsets cross-reacted with Delta and Omicron variants (44). Similarly, in the present study, we showed a significant increase in the frequency of IFN-γ-producing CD8^+^ and CD4^+^ T cells detected in the lungs of protected mice that received the pan-variant SARS-CoV-2 vaccine compared to non-protected mock-vaccinated mice. Overall, the vaccine was safe and immunogenic and provided cross-protection against multiple SARS-CoV-2 VOCs.

Interestingly, several earlier studies have performed epitope profiling in the existing COVID-19 mRNA vaccines (45, 46). One such study has mapped immunogenic amino acid motifs and linear epitopes of primary sequence of SARS-CoV-2 spike protein that induce IgG in recipients of PfizerBioNTech COVID-19 mRNA vaccine (45). The obtained data identified various distinctive amino acid motifs recognized by vaccine-elicited IgG, a subset of those recognized by IgG from natural infection (hospitalized COVID-19 patients), which can mimic three-dimensional conformation (mimotopes). The identified dominant linear epitopes in the C-terminal region of the spike protein are identical with those of SARS-CoV, bat coronavirus, and epitopes that trigger IgG during natural infection, but have limited homology to spike protein of non-pathogenic human coronavirus (45). In another study, high- resolution linear epitope profiling of recipients of Pfizer-BioNTech COVID-19 mRNA vaccine and COVID-19 patients was performed and found that vaccine-induced antibodies targeting viral spike RBD have a broader distribution across RBD than natural infection-induced antibodies (46). Moreover, mutation panel assays targeting the viral variants of concern demonstrated that the epitope variety induced by mRNA vaccine is rich in breadth, thus can grant resistance against viral evolutionary escapes in the future, which represents an advantage of vaccine-induced immunity (46). The identified epitopes in COVID-19 mRNA vaccine may form the basis for further research of immune escape, viral variants, and design of vaccine and therapy.

The SARS-CoV-2 virus shares homology to some degree at the level of its sequence, structure, and function with that of SARS-CoV and MERS-CoV (47). Studies revealed that anti SARS-CoV spike antibodies possessed the ability to inhibit the binding of SARS-CoV-2 to ACE2 (48). Furthermore, unique features of the spike, including crucial functions in host receptor binding, conserved sequences, good immunogenicity for neutralizing antibody induction, and effective target for T- cell responses, promote the priority of the spike for COVID-19 vaccine development. As per the WHO, among the existing clinical COVID-19 vaccine candidates, 32% (55/172) were recombinant protein vaccines. RNA vaccines, viral vectored vaccines, and inactivated virus vaccines accounted for 23% (40/172), 13% (23/172), and 13% (22/172), respectively. Except for the inactivated vaccine, most vaccine candidates are constituted by variations in antigen fragments from the spike. In brief, the antigen fragments may contain full length spike, different lengths of RBD, or even synthesized peptides as engineered multiepitope to induce high neutralizing antibodies to neutralize viruses (49, 50). Therefore, mutations in the spike of several SARS-CoV-2 variants impaired the protective efficacy of COVID-19 vaccines that were developed based on the spike.

Several COVID-19 vaccines have been approved for use and exert adequate protection when wild- type SARS-CoV-2 was still prevalent. NVX-CoV2373 from Novavax is a protein subunit vaccine showing 89.3% efficacy against SARS-CoV-2 in a phase III trial operated in United Kingdom (51). BNT162b2 from Pfizer-BioNTech and mRNA-1273 from Moderna are messenger RNA (mRNA) vaccines, showing 95% and 93.2% efficacy in preventing COVID-19 (28, 52). The adenovirus vector vaccine ChAdOx1 (AZD1222) also shows 90% efficacy if vaccinated at a low dose followed by a standard dose (53). In addition, the viral vectored and inactivated virus vaccines also exhibited effective protection against COVID-19 (53–55). However, these vaccine candidates are designed based on wild- type SARS-CoV-2, and the vaccine effectiveness against SARS-CoV-2 variants is of particular concern.

The effectiveness of the COVID-19 vaccine against Delta and Omicron variant transmission is a great concern. The effectiveness of BNT162b2 or mRNA-1273 vaccines in preventing contact transmission of Delta is reportedly between 9% and 38%. Even if fully vaccinated, the effectiveness is 27%–65% (56). The effectiveness of the ChAdOx1 vaccine against Delta transmission is 36%–42% (57). Notably, the protection of full vaccination against Omicron infection of close contacts was significantly reduced compared to that of Delta. The effectiveness of vaccination with COVID-19 mRNA vaccines against contact transmission decreased to 22.5% (58). These results indicate that physical protection, such as wearing a mask, is still necessary.

While looking at the effectiveness of currently authorized COVID-19 vaccines against symptomatic infection, hospitalization, and death induced by SARS-CoV-2 Alpha, Beta, Gamma, Delta, and Omicron variants, a decline in vaccine effectiveness was observed. The vaccine efficacy decreased to a different degree for each COVID-19 vaccine, even after full vaccination, especially for the protection of documented infection (59–63). The protective efficacy of the Novavax NVX-CoV2373 subunit vaccine declined from 89.3% to 49.4% in the clinical studies carried out in South Africa, where the Beta variant is prevalent (64). The effectiveness of the inactivated vaccine CoronaVac produced by Sinovac decreased to approximately 50% against symptomatic infection with the Gamma variant (65). BNT162b2 was reported with 95% efficacy against wild type in the clinical trial. However, in Qatar, the effectiveness of the BNT162b2 vaccine was reduced by approximately 20% against the Beta variant as detected 14 days after the second dose of vaccination (66). This is consistent with the speculation that the E484K mutation affects the efficacy of the BNT162b2 vaccine (67). Similarly, the efficacy of the ChAdOx1 chimpanzee adenoviral vector vaccine decreased to 10.4% against Beta variant (68). Other studies reported that the efficacy of one dose of the BNT162b2 vaccine was 33.5% and 51.1% against the Delta and Alpha variants, respectively (69). The ChAdOx1 vaccine also showed significant differences after one- or two-dose vaccinations against the Delta and Alpha variants. After the second dose, the efficacy elevated from 51.4% to 66.1% against the Alpha variant, while the protective efficacy increased from 32.9% to 59.8% against the Delta variant (70). In case of the Omicron variant, a marked reduction in the efficacy of most approved vaccines was observed. The antibody- neutralizing titers against Omicron in the serum collected from individuals with two doses of BNT162b2 were decreased more than 22- fold compared with that of the ancestor (71, 72). In addition, the sera from individuals fully vaccinated with ChAdOx1 vaccines could barely neutralize the Omicron variant (71). Later, several Omicron spike- specific vaccines were constructed. The results showed that boosting with mRNA-1273 had higher neutralizing antibody titers against Omicron than boosting with mRNA-Omicron (73).

Meanwhile, the Omicron S1 recombinant protein vaccine elicited a significantly weaker T-cell response compared to the prototype S1-based recombinant protein vaccine candidate (74), which indicates that the Omicron S1 subunit may not be an appropriate selection for developing specific vaccines against Omicron- included SARS-CoV-2 variants. Some other studies found that mRNA vaccines containing several pivotal mutations of the Omicron and Delta spike could elicit high neutralizing antibodies against the Omicron variant (75), suggesting the consideration of the essential mutations of SARS-CoV-2 virus spike, not limited to the currently prevalent VOCs. Taken together, the ongoing pandemic of SARS-CoV-2 variants calls for continuous surveillance of vaccine efficacy against emerging mutations, which is an extremely important factor for vaccine optimization.

In this context, universal pan-variant vaccines generating T- and B- cell-mediated immunity like that of ours will be of crucial help. Such vaccines could induce strong humoral and cellular immunity against several viruses. On the one hand, B cells are activated upon recognizing antigens with B- cell receptors (BCRs) and further differentiate into memory B cells or plasma cells that can produce antigen-specific antibodies. Th2 cells help in B- cell activation by secreting cytokines such as IL 4 and IL-5. The induced neutralizing antibodies can block viral infection at the initial stage. On the other hand, antigens from the combined vaccine are taken up, processed, and presented to CD4^+^ and CD8^+^ T cells by antigen-presenting cells (APCs) through MHCII and MHCI antigen complexes, respectively. Then, CD4^+^ T cells are activated and can differentiate toward Th1, Th2, and memory CD4^+^ T cells. Th1 cells also promote the activation of CD8^+^ T cells by generating cytokines such as IFN-γ, IFN-β, and IL-2. Activated CD8^+^ T cells can differentiate into effector and memory CD8^+^ T cells. The effector CD8^+^ T cells could kill and lyse infected cells by releasing cytokines, including IFN-γ.

Furthermore, the cross-reactive SARS-CoV-2-specific memory CD4^+^ and CD8^+^ T cells are present in up to 50% of unexposed, pre-pandemic, healthy individuals (UPPHI). However, the characteristics of cross-reactive memory CD4^+^ and CD8^+^ T cells associated with subsequent protection of asymptomatic COVID-19 patients (i.e., unvaccinated individuals who never develop any COVID-19 symptoms despite being infected with SARS-CoV-2) remains to be fully elucidated. Studies from our group and others have detected cross-reactive CD4^+^ and CD8^+^ T cells, directed toward specific sets of conserved SARS-CoV-2 epitopes, not only from unvaccinated COVID-19 patients but also from a significant proportion of unexposed pre-pandemic healthy individuals (UPPHI) who were never exposed to SARS-CoV-2 (33, 50, 76, 77). Cross-reactive SARS-CoV-2-specific memory CD4^+^ and CD8^+^ T cells are not only present in COVID-19 patients but also in up to 50% of UPPHI (50, 77–83). Moreover, pre-existing common cold coronavirus (CCCs)/SARS-CoV-2 cross-reactive memory CD4^+^ and CD8^+^ T cells are present in unvaccinated UPPHI who were never exposed to SARS-CoV-2 (33, 50, 76, 77, 84–89). These data suggest the presence of clones of memory CD4^+^ and CD8^+^ T cells in UPPHI induced following previous exposures with seasonal CCCs that cross-recognize conserved SARS-CoV-2 and CCCs epitopes (77, 90, 91). However, it is not yet known whether these cross-reactive memory CD4^+^ and CD8^+^ T cells: (i) preferentially cross-recognize the alpha CCCs (i.e., α-CCC-229E and α-CCC-NL63) or the beta CCCs (i.e., β-CCC-HKU1 and β-CCC-OC43); and (ii) the antigen-specificity, frequency, phenotype, and function of the cross-reactive memory CD4^+^ and CD8^+^ T cells associated with protection against COVID-19 severity in unvaccinated asymptomatic patients. Compared with unvaccinated severely ill COVID-19 patients and unvaccinated patients with fatal COVID-19 outcomes, unvaccinated asymptomatic COVID-19 patients displayed significantly (i) higher rate of the α-CCC strain 229E (α-CCC-229E); (ii) higher frequencies of functional memory CD134^+^CD137^+^CD4^+^ and CD134^+^CD137^+^CD8^+^ T cells directed toward cross-reactive α-CCCs/SARS-CoV-2 epitopes from structural, non-structural, and regulatory proteins; and (iii) lower frequencies of cross-reactive exhausted PD-1^+^TIM3^+^TIGIT^+^CTLA4^+^CD4^+^ and PD-1^+^TIM3^+^TIGIT^+^CTLA4^+^CD8^+^ T cells. These findings (i) support a crucial role of functional, poly-antigenic α-CCCs/SARS-CoV-2 cross-reactive memory CD4^+^ and CD8^+^ T cells, induced following previous exposures to α-CCC strains, in protection against subsequent severe disease caused by SARS-CoV-2 infection; and (ii) provides a strong rationale for the development of broadly protective, T-cell-based, multi-antigen universal pan- coronavirus vaccines.

Since the B- and T- cell epitopes used in this study are highly conserved between SARS-CoV-1 and MERS-CoV, it is likely that protection will be observed against these strains as well. However, providing direct evidence of protection induced by our multi-epitope vaccine against SARS-CoV-1 and MERS-CoV would require (i) generating a new DPP4/HLA-DR0101/HLA-A*0201 triple transgenic mouse model that needs backcrossing the DPP4 transgenic mice with our HLA-DR0101/HLA-A*0201 double transgenic mice, a process that is time consumable, as it will take several months to establish; and (ii) extensive *in vivo* and *in vitro* studies. Hence, demonstration of the breadth of protection induced by our multi-epitope vaccine against SARS-CoV-1 and MERS-CoV will be the subject of future independent reports in continuation to the existing study.

In conclusion, we report that a CoV vaccine targeting conserved B- and T- cell epitopes was safe, immunogenic, and provided cross-protection against six SARS-CoV-2 variants of concern, supporting the next-generation vaccine strategy.

## Data availability statement

The original contributions presented in the study are included in the article/**Supplementary Material**. Further inquiries can be directed to the corresponding author.

## Ethics statement

The studies involving humans were approved by University of California Irvine IRB committee. The studies were conducted in accordance with the local legislation and institutional requirements. The participants provided their written informed consent to participate in this study. The animal study was approved by The University of California-Irvine conformed to the Guide for the Care and Use of Laboratory Animals published by the US National Institute of Health (IACUC protocol # AUP-22-086). The study was conducted in accordance with the local legislation and institutional requirements.

## Author contributions

SP: Conceptualization, Data curation, Formal analysis, Investigation, Methodology, Project administration, Validation, Visualization, Writing – original draft, Writing – review & editing, Software. ND: Data curation, Formal analysis, Methodology, Project administration, Writing – original draft. LZ: Data curation, Formal analysis, Methodology, Project administration, Writing – original draft. II: Data curation, Writing – original draft. AQ: Data curation, Methodology, Formal analysis, Writing – original draft. PC: Data curation, Formal analysis, Methodology, Conceptualization, Investigation, Software, Validation, Writing – original draft. DT: Data curation, Writing – original draft. BS: Data curation, Writing – original draft. AS: Data curation, Writing – original draft. AC: Data curation, Writing – original draft. RE: Data curation, Writing – original draft. MS: Data curation, Methodology, Writing – original draft. HV: Data curation, Methodology, Writing – original draft. AN: Writing – original draft. BK: Writing – original draft. JU: Conceptualization, Writing – original draft. DG: Conceptualization, Writing – original draft. TJ: Conceptualization, Writing – original draft. LB: Conceptualization, Data curation, Formal analysis, Funding acquisition, Investigation, Methodology, Project administration, Resources, Validation, Visualization, Writing – original draft, Writing – review & editing.
