# Supplementary material for: Cross-protection induced by highly conserved human B, CD4+, and CD8+ T-cell epitopes-based vaccine against severe infection, disease, and death caused by multiple SARS-CoV-2 variants of concern
Source: Front Immunol. 2024 Jan 22;15:1328905. doi: 10.3389/fimmu.2024.1328905 (PMC10839970; doi:10.3389/fimmu.2024.1328905)
Supplement: Supplementary file 1 [file DataSheet_1.pdf]

Supplementary Material

Table of Content

Serial Number	Supplementary Material	Page
1	Supplementary Fig. S1	2
2	Supplementary Fig. S2	3
3	Supplementary Fig. S3	4
4	Supplementary Fig. S4	5

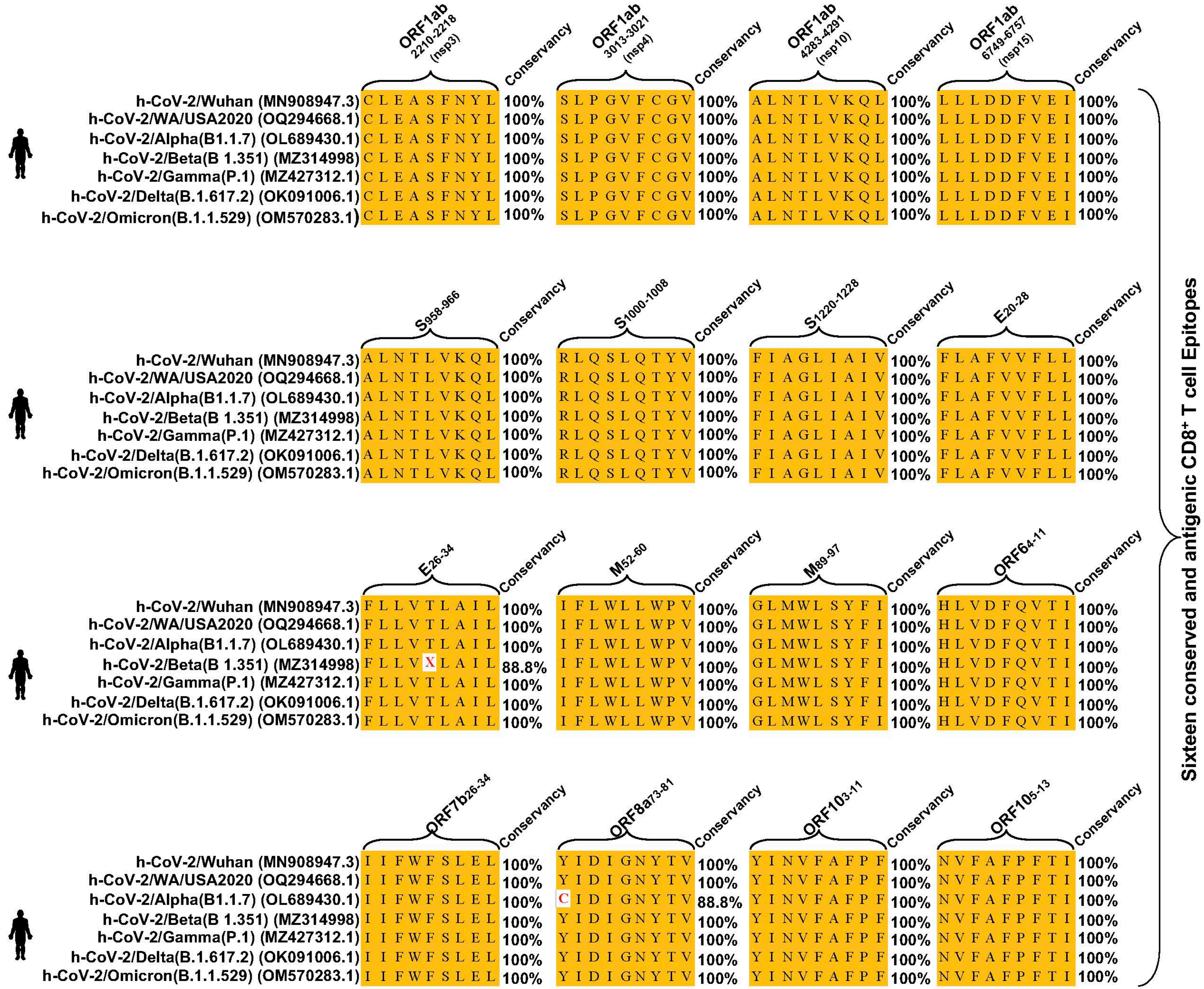

Supplementary Figure S1. Sequence homology analysis to identify the degree of the conservancy of the immunodominant CD8^{+} T cell epitopes among SARS-CoV-2 VOCs: Sequence homology data for the CD8^{+} T cell epitopes is shown. The 16 epitopes, found to be highly immunodominant against SARS-CoV-2 variants of concern WA/USA2020, Alpha (B.1.1.7), Beta (B.1.351), Gamma (P.1), Delta (B.1.617.2), and Omicron (B.1.1.529) were subjected to the sequence homology analysis.

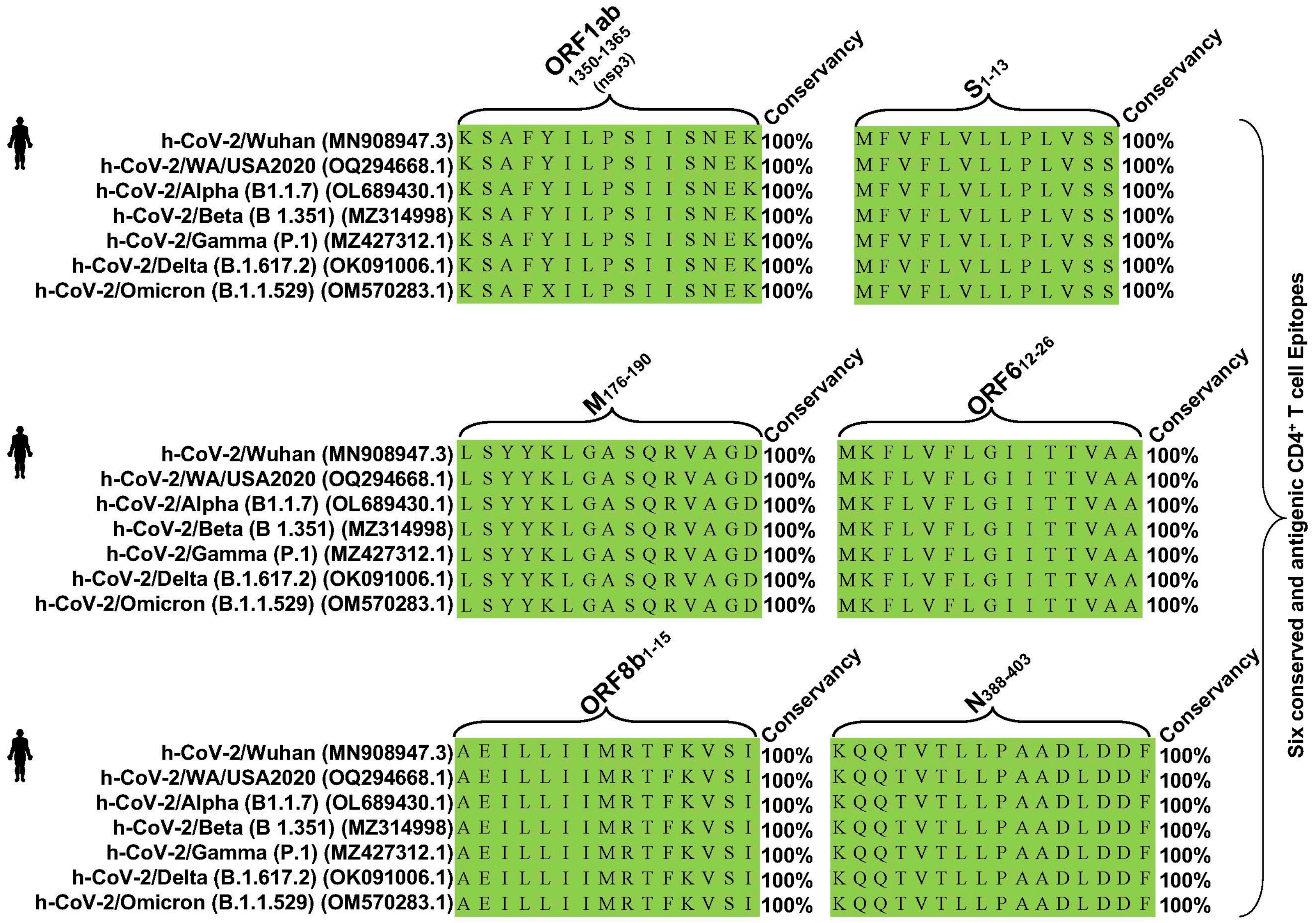

<u>Supplementary Figure S2</u>. Sequence homology analysis to identify the degree of the conservancy of the immunodominant $CD4^+$ T cell epitopes among SARS-CoV-2 variants of concern: Sequence homology data for the $CD4^+$ T cell epitopes is shown. The 6 epitopes, found to be highly immunodominant against SARS-CoV-2 variants of concern WA/USA2020, Alpha (B.1.1.7), Beta (B.1.351), Gamma (P.1), Delta (B.1.617.2), and Omicron (B.1.1.529) were subjected to the sequence homology analysis.

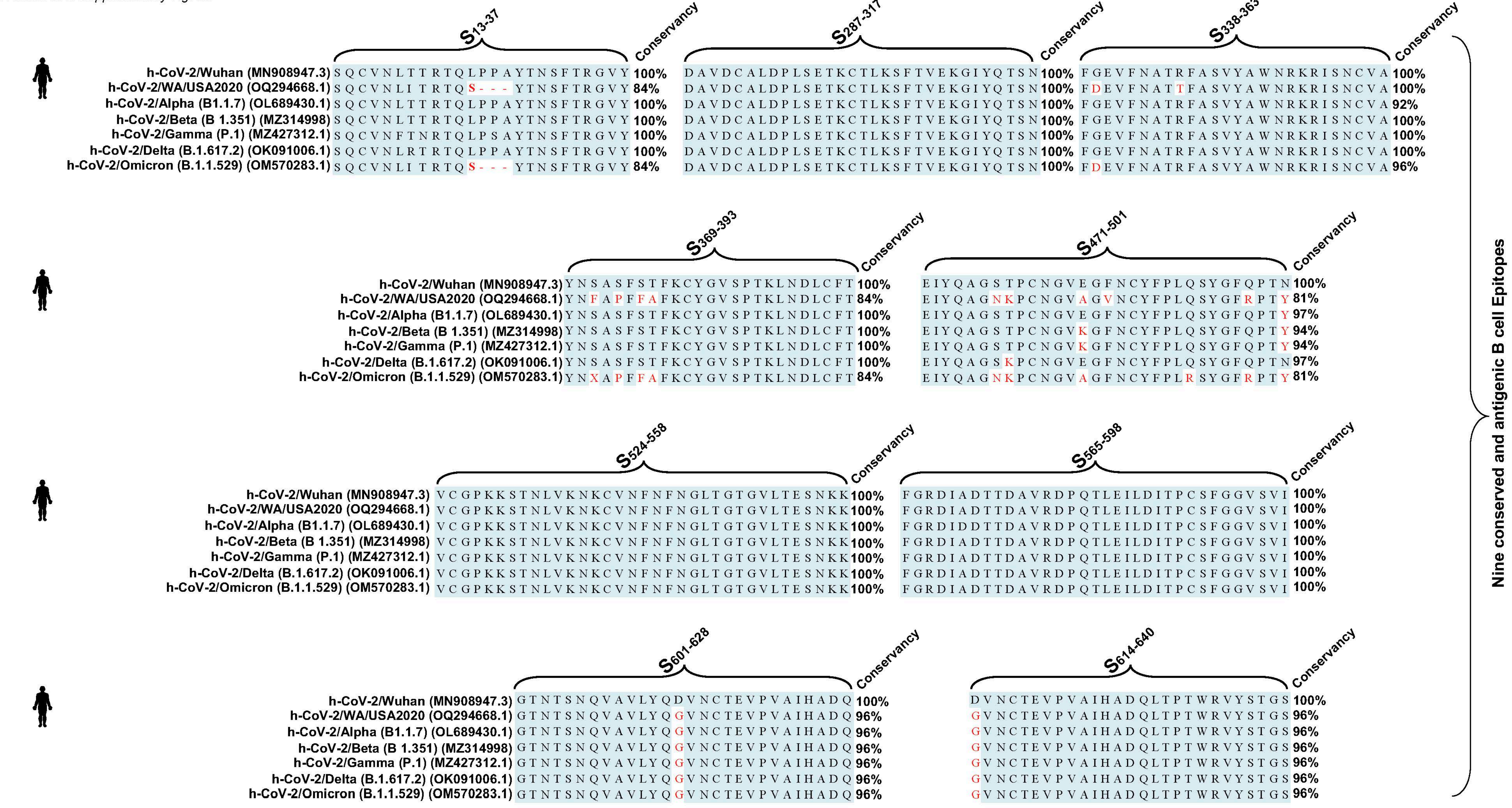

Supplementary Figure S3. Sequence homology analysis to identify the degree of the conservancy of the immunodominant B cell epitopes among SARS-CoV-2 variants of concern: The sequence homology data for the B cell epitopes are shown. The 9 epitopes, found to be highly immunodominant against SARS-CoV-VOCs WA/USA2020, Alpha (B.1.1.7), Beta (B.1.351), Gamma (P.1), Delta (B.1.617.2), and Omicron (B.1.1.529) were subjected to the sequence homology analysis.

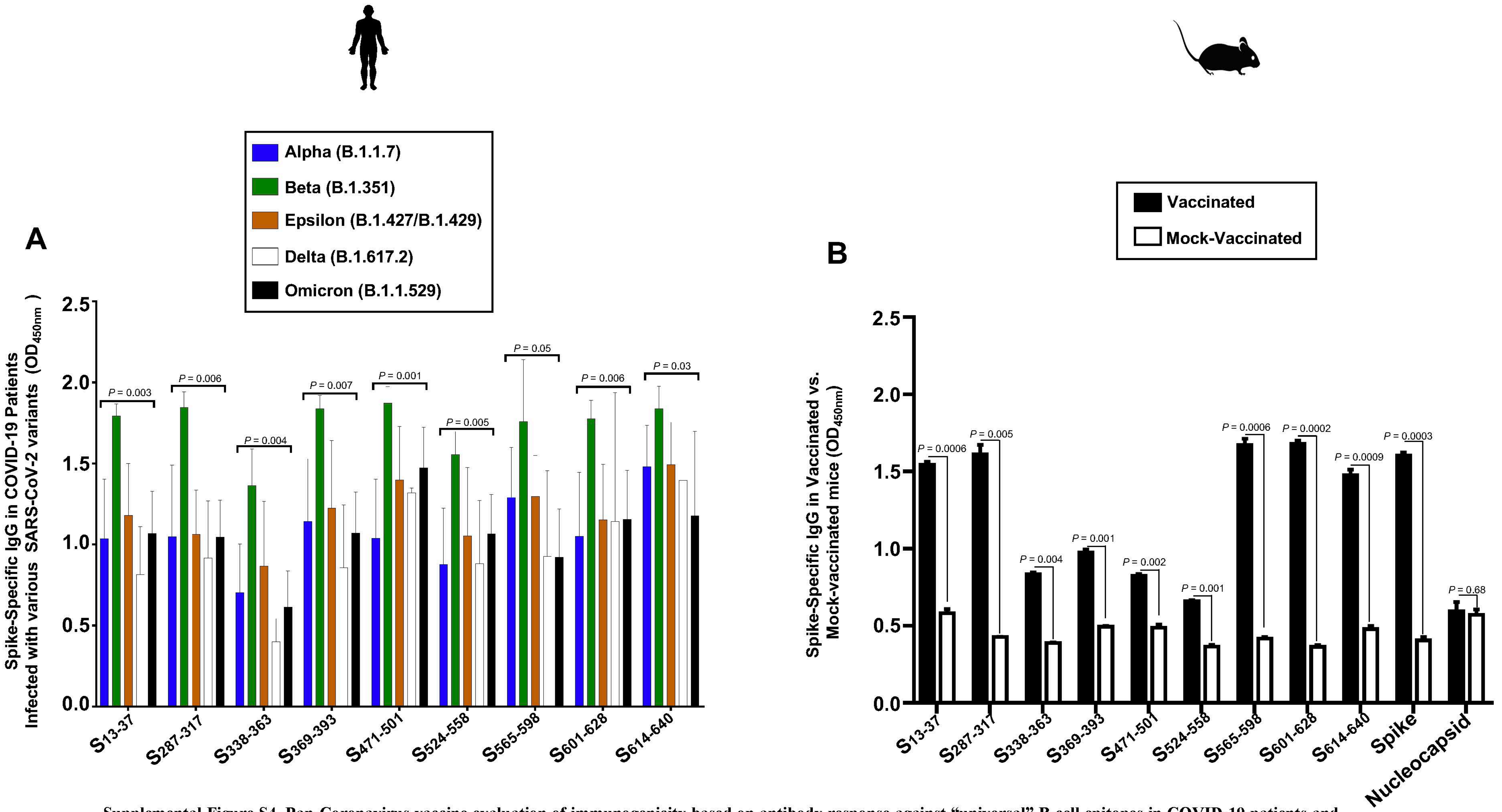

Supplemental Figure S4. Pan-Coronavirus vaccine evaluation of immunogenicity based on antibody response against "universal" B-cell epitopes in COVID-19 patients and triple transgenic HLA-A*02:01/HLA-DRB1*01:01-hACE-2 exposed to different SARS-CoV-2 variants of concern: Bar graphs show the peptide binding IgG level for the 9 "universal" B cell epitopes (A) among COVID-19 patients screened to be infected with SARS-CoV-2 variants of concern Alpha (B.1.1.7), Beta (B.1.351), Epsilon (B.1.427/B.1.429), Delta (B.1.617.2), and Omicron (B.1.1.529) and (B) in vaccinated versus mock-vaccinated triple transgenic HLA-A*02:01/HLA-DRB1*01:01-hACE-2 mice. Bars represent means ± SEM. Data were analyzed by student's *t*-test and multiple t-tests. Results were considered statistically significant at $P < 0.05$. Statistical correction for multiple comparisons was applied using the Holm-Sidak method.
